# A Jovian Magnetodisc Model for the Juno Era

**DOI:** 10.1029/2020JA028138

**Published:** 2020-09-25

**Authors:** J. E. P. Connerney, S. Timmins, M. Herceg, J. L. Joergensen

**Affiliations:** ^1^ Space Research Corporation Annapolis MD USA; ^2^ Nasa Goddard Space Flight Center Greenbelt MD USA; ^3^ Space Instrumentation Group Technical University of Denmark (DTU) Kongens Lyngby Denmark

**Keywords:** Jovian, magnetodisc, Juno spacecraft, Jupiter, magnetic field, magnetosphere

## Abstract

The Jovian magnetosphere assumes a disc‐like geometrical configuration (“magnetodisc”) owing to the persistent presence of a system of azimuthal currents circulating in a washer‐shaped volume aligned with, or near, the magnetic equatorial plane. A Voyager era empirical model of the magnetodisc is fitted to vector magnetic field measurements obtained during the Juno spacecraft's first 24 orbits. The best fitting (within 30 Jovian radii) magnetodisc model is characterized by an inner and outer radius of 7.8 and 51.4 Jovian radii, a half‐thickness of 3.6 Jovian radii, with a surface normal at 9.3° from the Jovigraphic pole and 204.2° System 3 west longitude. We supplement the magnetodisc model with a second current system, also confined to the magnetic equatorial plane, consisting of outward radial currents that presumably effect the transfer of angular momentum to outward flowing plasma. Allowing for variation of the magnetodisc's azimuthal and radial current systems from one 53‐day orbit to the next, we develop an index of magnetospheric activity that may be useful in interpretation of variations in auroral observations.

## Key Points

- An empirical magnetodisc model is fitted to the Juno magnetic field observations
- The magnetodisc model provides a more accurate representation of the magnetic field in the inner and middle magnetosphere of Jupiter
- The model is independently tested via observations of charged particle interactions with the Jovian satellites

## 1 Introduction

A planetary magnetosphere, as originally defined, is the region of space above the ionosphere within which the planet's magnetic field controls the motions of gas and fast charged particles (Gold, 1959). The term does not imply spherical geometry any more than the phrase “sphere of influence” does in politics. Nevertheless, when the first observations within the magnetosphere of Jupiter were obtained by the Pioneer 10 investigators, it was useful to introduce the term “magnetodisc” in reference to the confinement of plasma near the magnetic equator (Fillius & McIlwain, 1974; Northrop et al., 1974; Van Allen et al., 1974) and the magnetic field geometry implied by that confinement (Van Allen et al., 1974) and measured by the magnetometer (Smith et al., 1974). Confinement of Jovian plasma “to a region shaped like a discus” was anticipated (Gledhill, 1967) as a natural consequence of centrifugal force exerted on corotating plasma in a rapidly rotating dipolar magnetic field. That work was eventually extended to include nondipolar fields (Gleeson & Axford, 1976) and more realistic magnetohydrodynamic (MHD) equilibrium models (Caudal, 1986). However, it appears that centrifugal forces alone are insufficient to account for radial magnetic forces in the magnetodisc (Mauk & Krimigis, 1987; Mauk et al., 2004; Nichols et al., 2015), suggesting a consideration of particle pressure gradients and anisotropy for a more complete accounting.

After the Pioneer encounters, models of the magnetic field reflecting the magnetodisc geometry were constructed (Barish & Smith, 1975; Engle & Beard, 1980; Goertz, 1976) with emphasis on the more distant magnetosphere, fitted to Pioneer 10 observations. The Voyager encounters with Jupiter stimulated efforts to model the vector field in the inner and middle magnetosphere more accurately, leading to an axisymmetric model of the magnetodisc magnetic field that fit the Voyager 1 and 2 and Pioneer 10 observations very well within ~30 Jovian radii (Connerney et al., 1981, 1982). This empirical model employs a system of azimuthal (“ring”) currents circulating in a washer‐shaped volume in or near the magnetic equatorial plane; the current density within the magnetodisc varies as 1/*ρ* where *ρ* is the distance from Jupiter's magnetic dipole axis. The model is parameterized with an inner radius (*R*_0_), and outer radius (*R*_1_), disc half‐thickness (*D*), current density constant (*μ*_0_ *I*/2) and disc orientation (*θ*_*d*_, *ϕ*_*d*_). Analytical approximations valid throughout the magnetosphere (with negligible error except in the immediate vicinity of the inner edge, *R*_0_) were provided (Acuña et al., 1983; Connerney et al., 1981) to facilitate calculations, and subsequently, the model has found widespread application in studies of the gas giant magnetospheres (Balogh et al., 1992; Connerney et al., 1983; Connerney, Acuña, & Ness, 1996; Edwards et al., 2001; see, e.g., Pensionerov et al., 2019; Vogt et al., 2017).

The early Euler potential models of the magnetodisc (exemplified by Goertz, 1976) have been much improved in application to Galileo observations (Khurana, 1992, 1997); this type of model accommodates departures from axisymmetry and nonplanar current sheet geometries, allowing application throughout the magnetosphere. Likewise, comprehensive models including magnetopause and magnetotail currents, in addition to magnetodisc currents, have also been developed (Alexeev & Belenkaya, 2005) for the Jovian magnetosphere. This model used a magnetodisc current density that varies as 1/*ρ* ^2^ (another easily integrable form); however, Caudal's MHD equilibrium model suggests that a 1/*ρ* ^1.2^ variation in integrated current density would be more appropriate. Pensionerov et al. (2019) combined the model of Connerney et al. (1981) with that of Alexeev and Belenkaya (2005) in a piecewise fashion, also incorporating elements of Khurana's Euler potential models (Khurana, 1997) to more accurately model the field throughout a large volume of the Jovian magnetosphere. See Achilleos (2018) for a recent review of Jovian magnetodisc models.

In this note we confine our interest to the inner and middle magnetosphere (*r* < 30 *R*_*j*_) where axisymmetry holds reasonably well and simply fit Juno magnetic field observations acquired during the first 24 periapses to the original magnetodisc model of Connerney et al. (1981). Our intent is to provide a model that represents the measured field throughout Juno orbits well (past and future), and its time variation, without regard to the underlying physical processes. The equilibrium configuration of an outer planet magnetosphere is a rich subject (see, e.g., Achilleos et al., 2010; Achilleos et al., 2015; Achilleos, 2018; Bagenal & Delamere, 2011; Caudal, 1986; Kivelson, 2015; Nichols et al., 2015; Vogt et al., 2014) beyond the scope of this report.

Observations acquired during the very first Juno periapsis were sufficient to demonstrate that the Voyager era model parameters yielded a magnetodisc that stretched field lines outward along the equator a bit too much (Connerney, Adriani, et al., 2017). Juno mission planning and instrument sequencing has thus far used the Voyager era magnetodisc model (in combination with an internal field model), and we now have the opportunity to improve the model fit for the Juno era. We do extend the Voyager era model slightly, adding to the magnetodisc model another axisymmetric system of currents passing radially outwards within the magnetodisc; these currents are responsible for the transfer of angular momentum from Jupiter to outflowing plasma (Connerney, 1981b; Hill, 1979; Vasyliunas, 1983). The model radial currents are distributed uniformly in z within the magnetodisc and contribute to the azimuthal component of the magnetic field, simply computed using Ampere's law (Connerney, 1981b) and assuming axisymmetry.

The magnetodisc model is particularly important in establishing the size of the auroral zone and the ionospheric footprints of the spacecraft and Jovian satellites as a function of time. Juno traverses the polar regions very quickly, particularly now in the north, and sorting out where magnetic field lines map to (from near the pole to the equator) is challenging. We expect this revised model to do a much better job identifying observations associated with satellite interactions, as well as predicting future events that are of interest in instrument command sequencing. We also develop a periJove by periJove time series of magnetodisc and radial current intensity for the first 24 Juno periJoves in an effort to provide guidance on the time variability of these two systems, with an eye toward auroral dynamics.

## 2 Observations

The Juno spacecraft was inserted into polar orbit about Jupiter on 5 July 2016, with periJove of ~1.05 *R*_*j*_ (Jupiter radius, 1 *R*_*j*_ = 71,492 km) and apojove of ~113 *R*_*j*_. The Juno primary mission plan was designed (Bolton & the Juno Science Team, 2010; Bolton, Lunine, et al., 2017) to wrap the planet in a dense net of observations for mapping the planetary magnetic field, using periapsis passes spaced evenly in longitude about the planet. At the end of its primary mission phase, Juno will have completed 32 orbits separated by ~11° longitude at the equator. An extended mission, in the planning stage, would potentially double the number of periJoves, providing orbits separated by ~6° at the equator, and execute a targeted magnetic survey above the “Great Blue Spot” (Connerney et al., 2018; Moore et al., 2017). The spacecraft completes an orbit in about 53 days (Bolton, Adriani, et al., 2017), and the evolution of the orbit in time is best described as a rotation about the orbit plane that results in a northward drift of periapsis by about 1° latitude each periJove.

**Figure 1:**
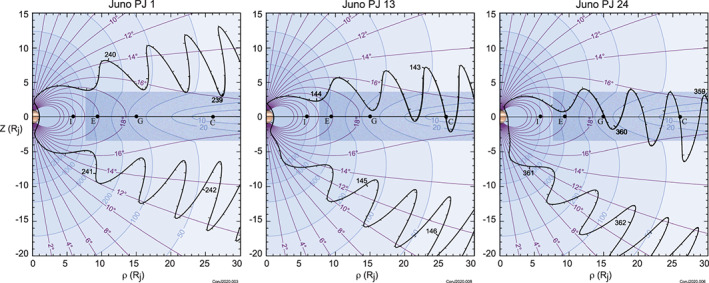
Evolution of Juno's trajectory in magnetic dipole coordinates calculated using the JRM09 model dipole coefficients. Model magnetic field lines (violet) are drawn every 2° in magnetic colatitude and contours of the model magnetic field magnitude are indicated from 20 to 2,000 nT (blue). The washer‐shaped disc within which the azimuthal (ring) currents flow (into the paper) is indicated by the shaded region. The radial distance to each of the Galilean satellites (Io, Europa, Ganymede, and Callisto) is indicated along the magnetic equator but in this coordinate system they oscillate up and down as the planet rotates due to the angular separation (10.3°) between the dipole and rotation axes.

The magnetodisc currents are sampled as the spacecraft penetrates into and passes through the current‐carrying region near the magnetic equator, as illustrated in Figure 1. Early in the mission, the spacecraft penetrated into the magnetodisc while inbound relatively distant (>30 *R*_*j*_) from the planet (Connerney, Adriani, et al., 2017). As such, the early orbits offered a relatively weak constraint on the distribution of current in the magnetodisc. Later orbits (see Figure 1) provided magnetodisc traversals in closer to Jupiter as a natural consequence of Juno's slow orbital evolution. In time, Juno will have sampled the magnetodisc throughout the entire region of interest (*r* < 30 *R*_*j*_) as magnetic equator crossings progress inward toward Jupiter. Traversals of the magnetodisc constrain the current as a function of radial distance (and height above the equator) very well, as can be appreciated by drawing a narrow Ampere's pillbox spanning the thickness of the magnetodisc at arbitrary radial distance (e.g., Acuña et al., 1983; Connerney et al., 1981). At this point in the mission (Orbit 24) we have sufficient coverage of the magnetodisc to significantly improve upon the Voyager era model, as applied to Juno era observations.

The vector magnetic field is measured continuously along Juno's trajectory by a pair of fluxgate magnetometers located at 10 and 12 m from the spacecraft's center on a magnetometer boom extending from one of Juno's three solar arrays (Connerney, Benn, et al., 2017). The field is sampled at a rate of 64, 32, or 16 samples per second depending on distance from Jupiter and telemetry allocations for a particular orbit. For this work we use samples of the field averaged over 60 s, corresponding to two rotations of the spacecraft about payload *z* axis, acquired within 30 *R*_*j*_ of the planet, in instrument dynamic range 0 (±1,600 nT, nominal, per axis). The instrument autonomously ranges up into Range 1 (±6,400 nT, per axis) as the field exceeds the range 0 limit on approach to Jupiter and returns to range 0 post‐periJove as the field declines to modest levels (Connerney, Benn, et al., 2017). The range 0 data are sufficient for magnetodisc modeling and restricting observations to range 0 avoids periods of higher magnetic field strength (>1,600 nT) dominated by the internal field. The range 0 magnetic field data have been archived on schedule and are available at the planetary data system (PDS).

## 3 Methods

We find the best fitting magnetodisc parameters over the first 24 orbits via simple least squares minimization allowing all six model parameters to vary, initializing the model with the parameters of the Voyager 1 model. We use the singular value decomposition (Lanczos, 1961) as described by Connerney (1981a) and Connerney et al. (1982) with the exception that we fix the internal field with the JRM09 degree 10 spherical harmonic model (Connerney et al., 2018). With 23 orbits of observations (Orbit 2 provided no useful science data as a result of a spacecraft anomaly and entry into safe mode), observations obtained every 60 s would produce, unnecessarily, a large matrix to invert. We decimated the data set to be inverted to one sample every 10 min, more than sufficient to follow the ~10‐hr periodicity in magnetodisc field that is observed in transit of the inner and middle magnetosphere.

In calculating the magnetic field due to the azimuthal currents, we have numerically evaluated the integrals provided in Connerney et al., 1981. In most applications, we (and others) have used the analytical approximations (Acuña et al., 1983; Connerney et al., 1981; see also Edwards et al., 2001), which do a good job approximating the field for positions removed from the inner edge of the magnetodisc current‐carrying region. However, Juno traverses the inner edge of the magnetodisc current sheet where the approximations do a poor job; use of the approximations in that area produces an unsightly discontinuity across the inner edge of the magnetodisc.

**Figure 2:**
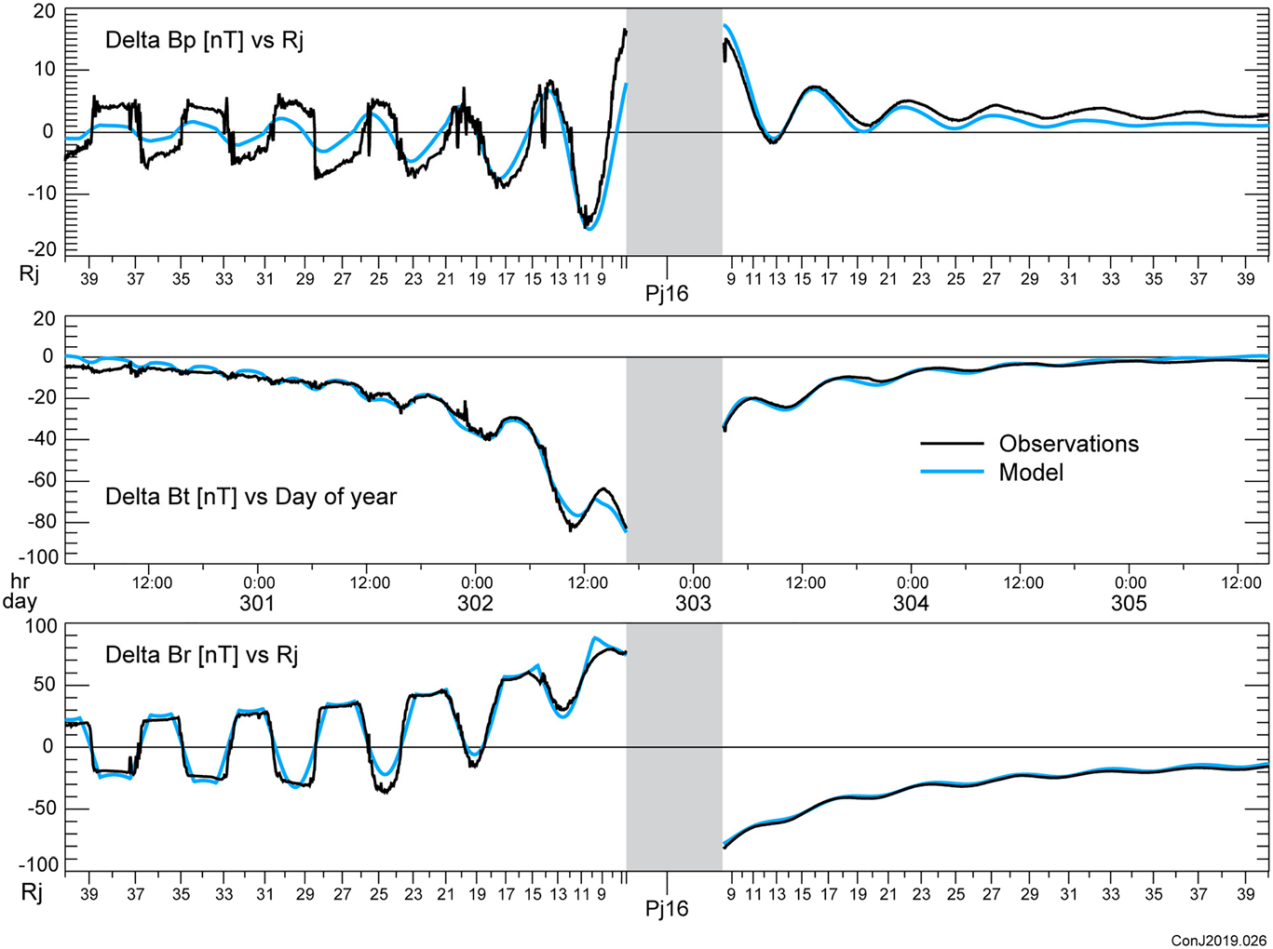
Perturbation plot for Periapsis Pass 16 illustrating a comparison of the observed vector magnetic field (after removal of the internal field using the JRM09 model) and the modeled (*r* < 30 *R*_*j*_) magnetodisc field, using the model fit parameters listed in Table 1. From top to bottom we plot the azimuthal, theta, and radial components of the observed (black) and modeled (blue) field. The shaded region represents a strong field region (>1,600 nT) dominated by the planetary field and excluded from consideration. Periapais 16 is an example of a pass fitting the azimuthal component poorly inbound to periJove, while fitting the theta and radial components well. The outbound portion of the periapsis pass has a distinctly different character as a result of an outbound trajectory at higher magnetic latitudes, remaining well above the current‐carrying region.

**Figure 3:**
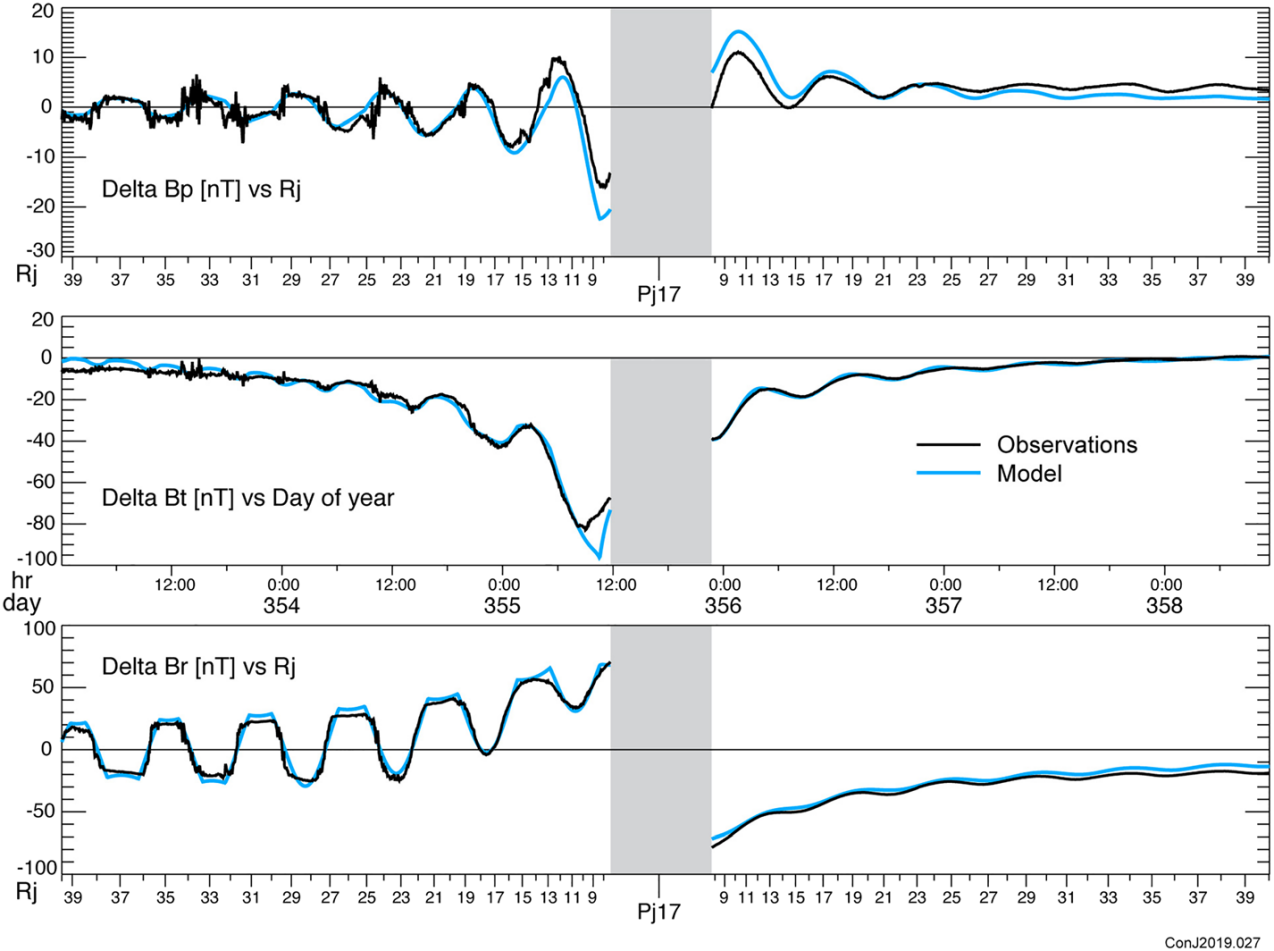
Perturbation plot for Periapsis Pass 17 as in Figure 2, illustrating a reasonably good fit to all three components.

**Figure 4:**
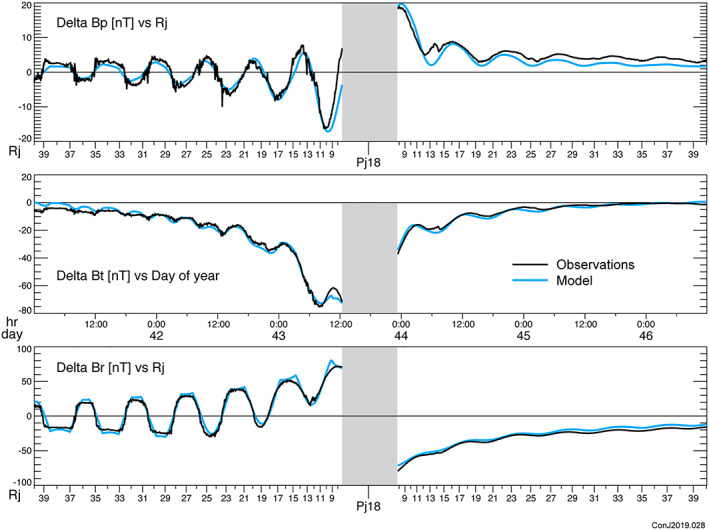
Perturbation plot for Periapsis Pass 18 as in Figure 2, also illustrating a reasonably good fit to all three components.

A model solution (Table 1) is constructed iteratively by summation over the independent eigenvectors of parameter space, but as we solve for all 6 free parameters of the magnetodisc, any least squares minimization ought to yield the same result. A comparison between the observed magnetic field and that calculated from the model is conveniently presented in the form of a “perturbation plot.” To isolate the field produced by currents external to the planet, we remove an estimate of the planetary field, for which the JRM09 spherical harmonic model is used, and the components of the vector field remaining (and attributed to external currents) are presented for comparison with model computations. A few representative periapsis passes are illustrated in Figures 2, 3, 4, and similar plots of all of the periapsis passes available to date are available in the supporting information. In plotting the model and data in Figures 2, 3, 4, we have extended the comparison beyond the fit interval (<30 *R*_*j*_) to provide some additional insight into the behavior of the model beyond our fit interval. We have also used 1‐s averages of the magnetic field for comparison with the model to provide better visibility into the time variability of the field.

**Table 1:** Magnetodisc Parameters

Parameter	Value	Description	Units
*R*_0_	7.8	Disc inner radius	Jovian radii
*R*_1_	51.4	Disc outer radius	Jovian radii
*D*	3.6	Half thickness	Jovian radii
*μ*_0_ *I*/2	139.6	Current constant	nT
*θ*_*D*_	9.3	Disc normal from rotation axis	degrees
*φ*_*D*_	204.2	Azimuth angle of disc normal	degrees

This model fits *all* of the vector magnetic field observations acquired by Juno within 30 *R*_*j*_ (Range 0 data only, Periapsis Passes 1–24) with a root‐mean‐square (RMS) residual of 4.2 nT. This set of model parameters does fit the Juno observations significantly better than those of the 1981 model, which fit Juno observations with an RMS residual of 8.7 nT. Nevertheless, the goodness of fit is probably best judged by inspection of the plots and compared to random fluctuations of the field. Some periapsis passes are of course fit better than others; the best fitting periapsis pass (PJ3) has an RMS residual of 2.4 nT and the periapsis fitting most poorly (PJ22) has an RMS residual of 5.9 nT. Other authors report RMS residuals over different radial distance intervals, making direct comparison difficult, but the residual quoted here is in family with that found by Pensionerov et al. (2019) fitting Juno PeriJove 1 observations between 15 and 40 *R*_*j*_. Vogt et al. (2017) fit the Connerney et al. (1981) model to 31 Galileo orbits, allowing the current constant to vary, with comparable RMS residuals over their fitted radial range (10 to 30 *R*_*j*_). The Voyager 1 magnetic field was fit with an RMS of 7.8 nT inside of 20 *R*_*j*_ (Connerney et al., 1982), but this fit included variation of the internal magnetic field as well and the fit interval extended slightly closer to Jupiter than the current data set.

**Figure 5:**
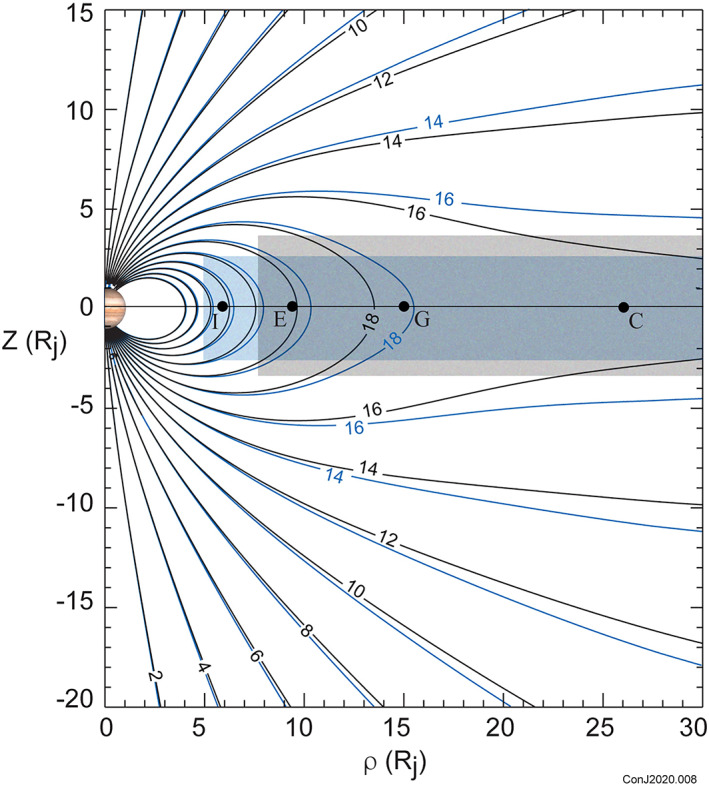
Comparison between the Jovian magnetic field geometry resulting from the magnetodisc model fit to Juno observations (black) and that fit to the Pioneer and Voyager flybys (blue). The shaded regions indicate the spatial extent of the washer‐shaped region carrying azimuthal current in each model (gray, Juno era; blue, Pioneer/Voyager).

The magnetodisc geometry resulting from the Juno era model (parameters in Table 1) is compared with that of the original model fitted to the Pioneer and Voyager flybys (Connerney et al., 1981) in Figure 5. The geometry of field lines in the Juno era model is comparable to that of the earlier model, but the Juno era model field lines are less extended (“stretched”) outward along the magnetic equator, as anticipated. Bearing in mind the rather unphysical geometry imposed on the current‐carrying region illustrated in half‐tones for the two models, one ought not attach much significance to the differences between them. Both are clearly poor representations of the actual extent of azimuthal currents particularly in the region near the inner edge of the model disc. Examination of the many perturbation plots provided in the supporting information suggests that the magnetodisc thickness decreases with increasing radius, at least for some periJoves, and that the radial currents are sometimes (e.g., PJ16 illustrated in Figure 2) more closely confined to the center of the magnetodisc than during other times. This is suggested by the rapid transition from positive to negative B∅ in evidence inbound toward periapsis.

**Figure 6:**
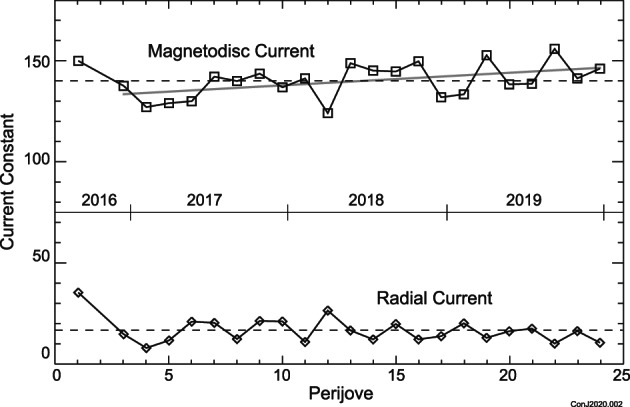
Time variability of the magnetodisc and radial current intensities obtained in fitting data from each Juno periJove independently. Dashed lines indicate average values (139.6 and 16.7 respectively) and the half‐tone line represents a linear fit to magnetodisc currents PJ3–PJ24.

Having found a model magnetodisc that minimizes the mean squared difference from the data acquired across all 24 periapsis passes, we now look at each individual periapsis pass to assess time variability of the current systems. We adopt as fixed the geometrical parameters listed in Table 1 and seek the best fitting model for each periapsis pass, independently, allowing the magnetodisc current constant (*μ*_0_ *I*/2) to vary, along with that of the radial current system (*μ*_0_ *I*_rad_/2*π*). In both cases, following prior usage, we list proportionality constants in nT and normalize all distances to planet radius, 71,492 km. Figure 6 shows the variation of both as a function of periJove number (time); tabulated values are provided in Table 2, along with the rms residual of the fit for each periapsis pass. Allowing variation of the magnetodisc and radial current systems from periapsis to periapsis improves the fit to each (PJ4, with a rms residual of 2.2 nT, replaces PJ3 as the one with the best fit; PJ22 at 4.6 nT remains the worst).

**Table 2:** Magnetodisc and Radial Current Fitted to Individual PeriJoves

PeriJove	Year	DOY	*μ*_0_ *I*_MD_/2	*μ*_0_ *I*_R_/2*π*	rms
1	2016	240	150.1	35.2	4.4
3	2016	346	137.8	14.6	2.3
4	2017	33	127.2	7.7	2.2
5	2017	86	129.1	11.5	2.8
6	2017	139	130.1	20.8	3.2
7	2017	192	142.3	20.2	3.6
8	2017	244	140.1	12.2	3.5
9	2017	297	143.8	21.1	3.6
10	2017	350	137.0	20.9	3.7
11	2018	38	141.4	10.7	3.0
12	2018	91	124.2	26.3	3.8
13	2018	144	148.9	16.4	3.8
14	2018	197	145.3	12.0	3.1
15	2018	250	144.8	19.6	3.6
16	2018	302	149.9	12.0	3.5
17	2018	355	132.1	13.6	3.0
18	2019	43	133.5	20.0	2.8
19	2019	96	152.9	12.8	3.8
20	2019	149	138.5	16.0	3.2
21	2019	202	138.8	17.3	3.8
22	2019	255	156.1	9.9	4.6
23	2019	307	141.4	16.1	3.3
24	2019	360	146.3	10.3	4.2

## 4 Results

Figure 6 suggests that the azimuthal current system responsible for the magnetodisc geometry is relatively stable, at least over the time spanned by Juno's first 24 orbits. Variations in time are but a few percent of the current intensity (mean ~140.2; standard deviation ~8.8) and all of the periJoves are fit reasonably well particularly in the radial and theta components of the field. The height‐integrated current (product of current constant and half‐thickness, *D*, in *R*_*j*_, of ~505.) as a function of radial distance appears to be about 12–15% less than that observed during the Pioneer and Voyager era. These results may also be directly compared with Vogt et al.'s (2017) extensive study of the Galileo era magnetodisc (using the same model) that yielded a height‐integrated current constant of ~530 in 1996 and ~645 in 1997 (normalized in the same way).

The variation in magnetodisc and radial currents illustrated in Figure 6 do not show, by themselves, a convincing trend in local time, which varies systematically with time, with orbit 1 near the dawn meridian and Orbit 24 just after midnight. However, it is worth noting that both Khurana (2001) and Vogt et al. (2017) identified a modest local time variation of magnetodisc currents within 30 *R*_*j*_ radial distance, with azimuthal currents strongest near midnight (tailward) and weakest near noon. PeriJove 1 appears a bit of an outlier in Figure 6, and omitting that measurement, the Juno observations are consistent with a modest increase in magnetodisc current as the orbit evolved from dawn to midnight. The linear fit is *μ*_0_ *I*_MD_/2 = 131.66 + 0.623 × pj, where pj is the orbit sequence from 3 to 24. The linear fit's constant has a standard deviation of 3.68, and the slope standard deviation is 0.25, indicating a significant trend in agreement with the work of Khurana (2001) and Vogt et al. (2017). The linear fit suggests a magnetodisc current constant of ~131 near dawn increasing to ~146 near midnight, and corresponding height‐integrated values of 471 and 525, respectively.

In contrast, the radial current system contributing to the azimuthal magnetic field varies significantly from periJove to periJove (mean ~16.7; standard deviation ~6.4), and while some periJoves (e.g., PJ17 and PJ18) are fit reasonably well by the model, several (like PJ16) are not, evidencing a more dynamic variation (supporting information). This outward radial current is of course the net current supplied to the magnetodisc from each polar region, and there is no reason to assume that both polar regions supply the same net current, as we have here for simplicity. Indeed, Kotsiaros et al. (2019) mapped Birkeland currents in both polar regions and found significantly more current flowing into and out of the southern auroral oval. But if we simply sum both polar regions, using the average value obtained in the fit, the outward radial current flowing in the magnetodisc during this time is ~12 × 10^6^ A.

It is plausible that the variation of *radial* current described here is diagnostic of the transport of angular momentum to outflowing plasma, and as such a barometer of Jovian magnetospheric dynamics. Several Juno mission primary magnetospheric objectives are related to the relationship between variations in auroral intensities, angular momentum transport, and solar wind variations (Bagenal et al., 2017), for which this measure of activity may prove useful. The record in Figure 6 is far from continuous, with just a few days sampled every 53 days or so, and the record of auroral observations is even less so; nevertheless, there is a growing body of work linking variations in solar wind ram pressure to variations in auroral intensities (Baron et al., 1996; Clarke et al., 2009; Connerney Satoh, & Baron, 1996; Gurnett et al., 2002; Kita et al., 2019; Murakami et al., 2016; Nichols et al., 2017; Tao et al., 2016) and radio emissions (Barrow et al., 1986; Desch & Barrow, 1984; Galopeau & Boudjada, 2005; Hess et al., 2012; Ladreiter & Leblanc, 1989; Louarn et al., 2001; Prangé et al., 1993; Terasawa et al., 1978). However, Jupiter's magnetosphere is driven both by internal processes and solar wind ram pressure variations (Vogt et al., 2019).

**Figure 7:**
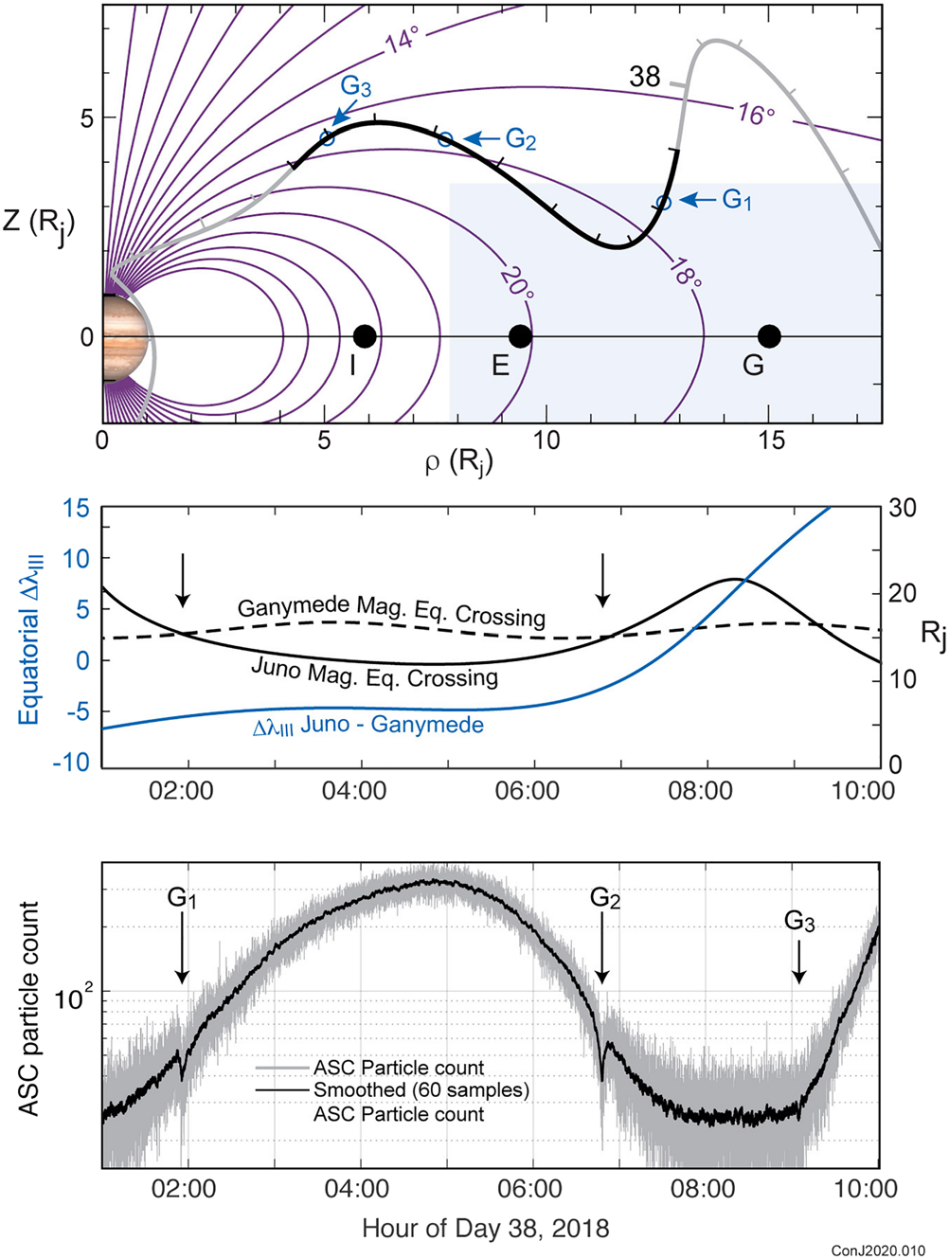
Energetic electron signatures associated with the interaction between Ganymede and the Jovian magnetosphere observed during Juno's approach to periapsis (PeriJove 11). The top panel illustrates the geometry of the observations in magnetic coordinates. Juno's trajectory is illustrated in this coordinate system in gray and black; the black section from hour 1 on day 38 through hour 10 corresponds to the path traversed by the spacecraft during the observations (energetic electron counts) shown in the bottom panel. The light blue shaded region indicates the spatial extent of the washer‐shaped region carrying azimuthal current in this model. The center panel shows the variation in radial distance to the magnetic equator for field lines crossed by Juno and the satellite Ganymede, and the separation in azimuth of those points, as a function of time.

The model magnetic field geometry can be independently tested using charged particle signatures associated with known satellites, at times when spacecraft and satellite are sampling the same particle population. The satellite may act as a source or sink (or both) of particles, depending on the nature of the interaction, and present as either a precipitous drop or increase in particle fluxes. In Figure 7 we present observations obtained by Juno during periJove 11 showing multiple absorption signatures due to Ganymede resulting from a particularly auspicious alignment of satellite and spacecraft. The particle flux being monitored in this example is the count rate of CCD pixels not optically excited, presumably due to energetic electrons (*E* > 10 MeV) penetrating the shielding of the Advanced Stellar Compass (ASC) Camera Head Unit (CHU). The ASC (Connerney, Benn, et al., 2017) is configured to record the number of pixels in each image (0.25‐s integration) excited by penetrating radiation, creating a nearly continuous time series as Juno passes through the radiation environment (Becker, Alexander, et al., 2017; Becker, Santos‐Costa, et al., 2017). Pixels excited by radiation appear in isolation, thereby distinguished from optical sources that excite a group of pixels within the CHU's optical point spread function.

The top panel of Figure 7 illustrates the geometry of the observations and demonstrates the utility of satellite interaction signatures in tracing field lines across great distances. Juno's trajectory in magnetic coordinates provided several instances during which spacecraft and satellite sampled the same population. Conservation of the first adiabatic invariant ensures that his occurs when both are crossing field lines with the same equatorial magnetic field strength (|*B*|_eq_). In this example, Juno and the satellite have |*B*|_eq_ within a few degrees of longitude of each other. Thus, it appears that the spacecraft and the satellite may have remained in close proximity, magnetically, throughout much of Juno's inbound transit. In Table 3, we compare the times at which satellite signatures were observed with those predicted using the model magnetic field to calculate |*B*|_eq_ as a function of time for both spacecraft and satellite.

**Table 3:** Satellite Interaction Signatures

PJ	Year	DOY	Satellite	Time of event	Predict time of event
11	2018	38	Ganymede	38–01:55:30	38–01:53:24
11	2018	38	Ganymede	38–06:48:00	38–06:53:40
11	2018	38	Ganymede	38–09:10:30	38–09:15:00
13	2018	144	Europa	144–05:09:35	144–05:09:30
18	2019	43	Europa	43–12:56:00	43–12:58:58
23	2019	307	Europa	307–23:18:40	307–23:18:40

**Figure 8:**
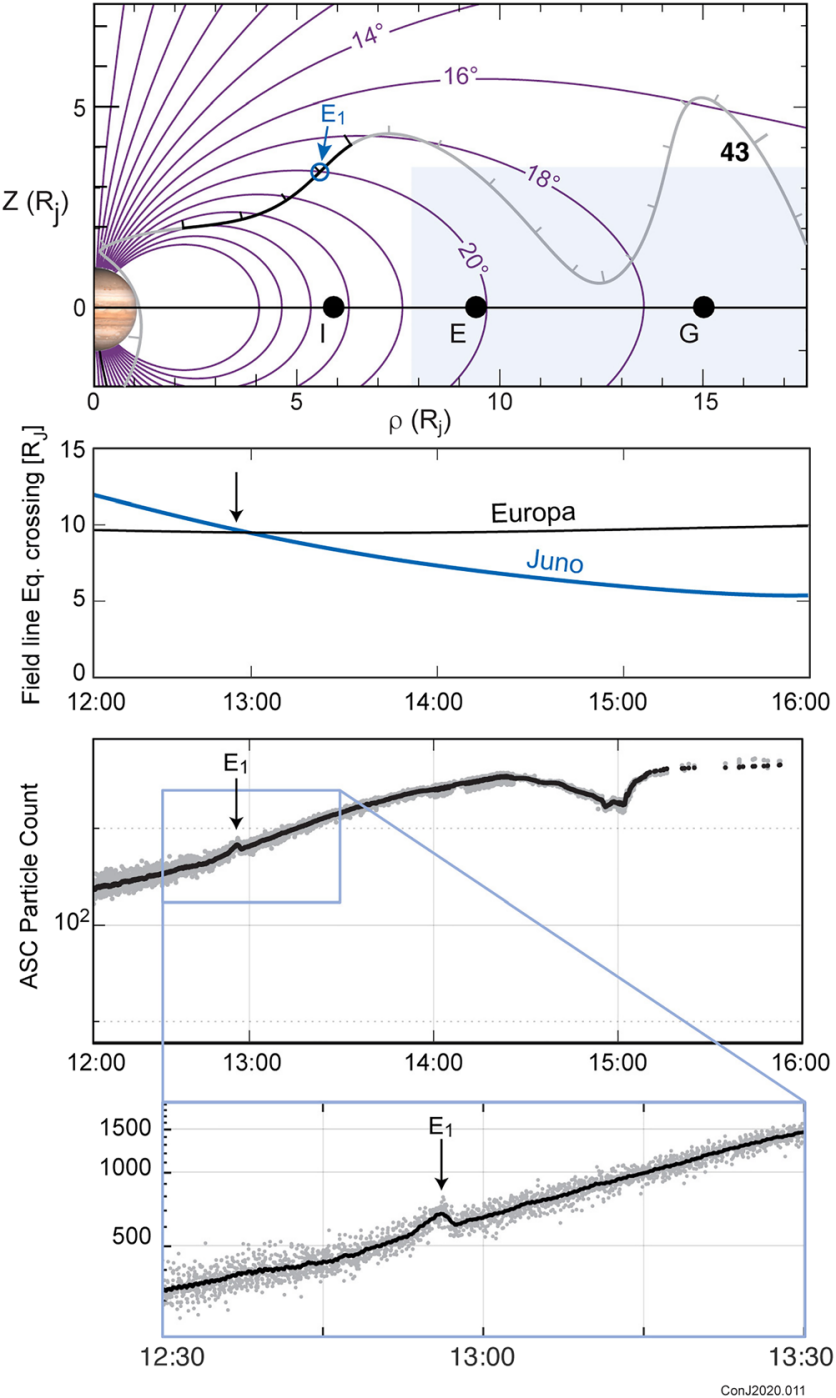
Energetic electron signatures associated with the interaction between Europa and the Jovian magnetosphere observed during Juno's approach to periapsis (PeriJove 18). The top panel illustrates the geometry of the observations in magnetic coordinates. Juno's trajectory is illustrated in this coordinate system in gray and black; the black section from hour 12 through hour 16 on day 43 corresponds to the path traversed by the spacecraft during the observations (energetic electron counts) shown in the bottom two panels. The center panel shows the variation in radial distance to the magnetic equator for field lines crossed by Juno and the satellite Europa, as a function of time. Separation between the two in longitude was near 0 throughout the interval. The more complex, broadened signature at ~15:00 is due to interaction with the satellite Io and the Io torus.

The satellite Europa provided three instances of close longitudinal alignment during periJoves 13, 18, and 23. One of these (PJ18) occurred while Juno was relatively far from periapsis, as illustrated in Figure 8. The other two instances occurred while Juno was in a strong magnetic field just above Jupiter's poles. The examples presented in Table 3 indicate that the model does very well in predicting the occurrence of these events in some instances, and less well in others. In particular, we note that the model does a very good job in predicting the Europa signatures observed during PJ 13 and 23, where the model traces the path of a magnetic field line from very near Jupiter's poles out to the orbit of Europa; it does less well when tracing the path of a field line from an intermediate position, as in Figure 8, near the magnetodisc's inner edge. This may reflect the nonphysical geometry of the model at its inner edge. One might imagine that the error between the model and the actual field accumulates in a bipolar fashion as one moves outward along the field in the vicinity of its inner edge. Tracing field lines only part of the way across this poorly modeled region may yield a larger discrepancy in the timing of satellite interaction events, not benefiting from cancelation of errors traced along the entire path.

## 5 Conclusions

We have revised the parameters of a simple, empirical magnetodisc model that is often used to describe the magnetic field in the inner and middle magnetosphere of Jupiter. It represents a significant improvement upon the existing model and offers an assessment of the time variability of magnetodisc azimuthal and radial current systems. While the magnetodisc appears remarkable stable, orbit by orbit fits demonstrate dynamic behavior of the magnetosphere, including occasional thinning of the thickness of the magnetodisc with increasing radius, and variation in radial current intensity. The observation of particle signatures (e.g., energetic electron fluxes) associated with Jovian satellites provides an opportunity to independently test the accuracy of modeled magnetic field geometry.

## Conflict of Interest

The authors are not aware of any real or perceived conflicts of interest with respect to the results of this paper.

## Supporting information

Supporting Information S1

## Data Availability

Data supporting the conclusions in this paper are available at the NASA Planetary Data System (https://pds.nasa.gov) (https://pds-ppi.igpp.ucla.edu/search/view/?f=yes&id=pds://PPI/JNO-J-3-FGM-CAL-V1.0/DATA/JUPITER/PC).
