# Supplementary material for: A Jovian Magnetodisc Model for the Juno Era
Source: J Geophys Res Space Phys. 2020 Sep 25;125(10):e2020JA028138. doi: 10.1029/2020JA028138 (PMC7583372; doi:10.1029/2020JA028138)
Supplement: Supplementary file 1 — Supporting Information S1 [file JGRA-125-e2020JA028138-s001.pdf]

AGU PUBLICATIONS

Journal of Geophysical Research – Space Physics

Supporting information for

A Jovian Magnetodisc Model for the Juno Era

J. E. P. Connerney[1,2], S. Timmins[2], M. Herceg[3], and J. L. Joergensen[3]

[1]Space Research Corporation, Annapolis, MD 21403

[2]Nasa Goddard Space Flight Center, Greenbelt, MD 20771

[3]Technical University of Denmark (DTU), Kongens Lyngby, 2800, Denmark

Correspondence to: Jack.Connerney@nasa.gov

Contents of this file:

Figs. S1 to S23
Table S1

Additional Supporting Information (Files uploaded separately)

Not Applicable

Figures S1-S23: Perturbation plots for Juno periapsis passes 1-24 (PeriJoves PJ 1, PJ3-PJ24), illustrating a comparison of the observed vector magnetic field (after removal of the internal field using the JRM09 model) and the modeled (r<30 R_j) magnetodisc field, using the model fit parameters listed in Table 1. From top to bottom we plot the azimuthal, theta, and radial components of the observed (black) and modeled (blue) field. The shaded region represents a strong field region (>1600 nT) dominated by the planetary field and excluded from consideration.

Table 1: Magnetodisc Parameters

Parameter	Value	Description	Units
**R0**	7.8	Disc inner radius	Jovian radii
**R1**	51.4	Disc outer radius	Jovian radii
**D**	3.6	Half thickness	Jovian radii
$\mu_0 I/2$	139.6	Current constant	nT
θ_D	9.3	Disc normal from rotation axis	degrees
φ_D	204.2	Azimuth angle of disc normal	degrees

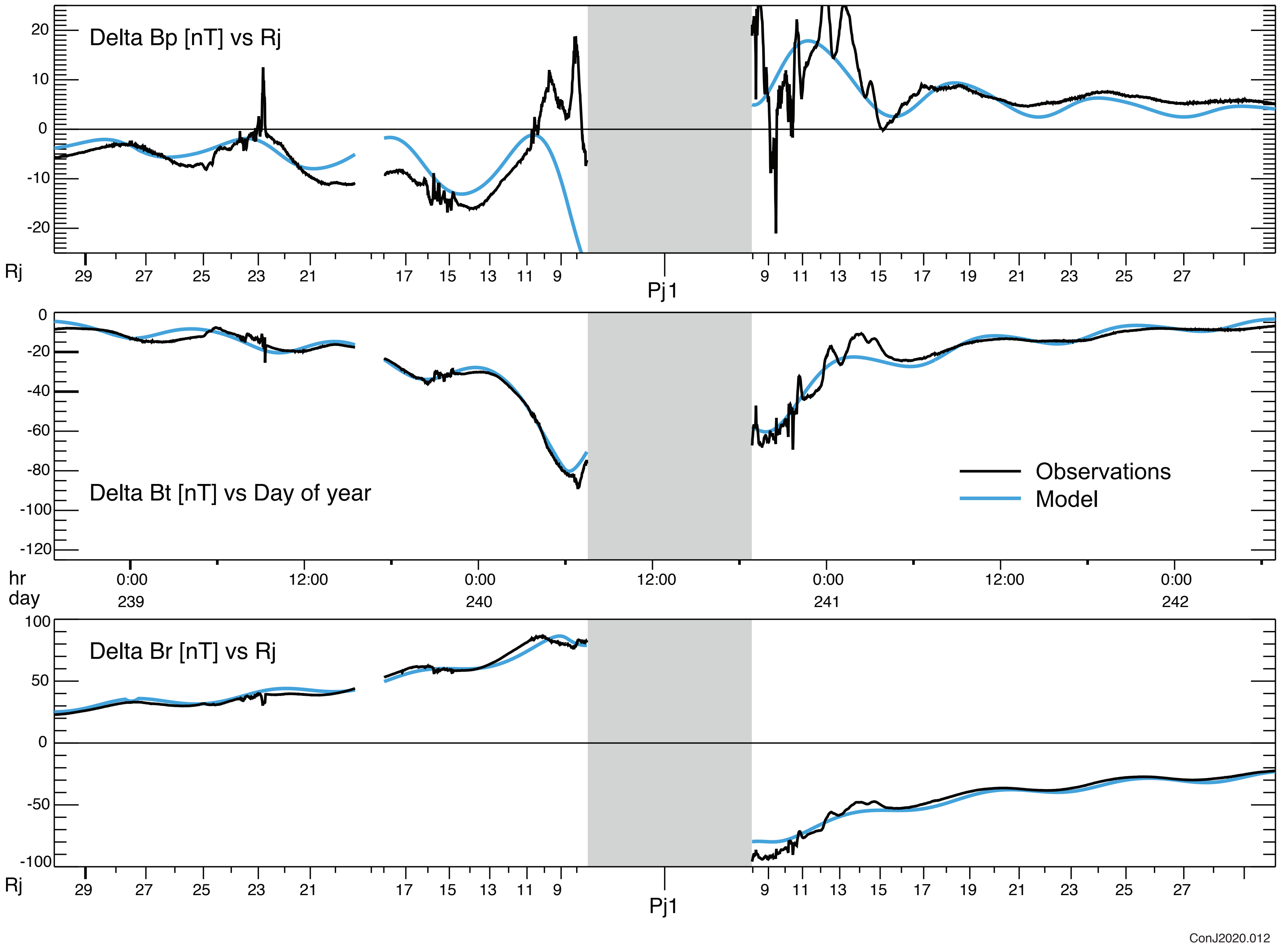

Delta Bp [nT] vs Rj
20
10
0
-10
-20
Rj
29 27 25 23 21 17 15 13 11 9
Pj1
9 11 13 15 17 19 21 23 25 27
Delta Bt [nT] vs Day of year
0
-20
-40
-60
-80
-100
-120
Observations
Model
hr
day
0:00 12:00 0:00 12:00 0:00 12:00 0:00
239 240 241 242
Delta Br [nT] vs Rj
100
50
0
-50
-100
Rj
29 27 25 23 21 17 15 13 11 9
Pj1
9 11 13 15 17 19 21 23 25 27
ConJ2020.012

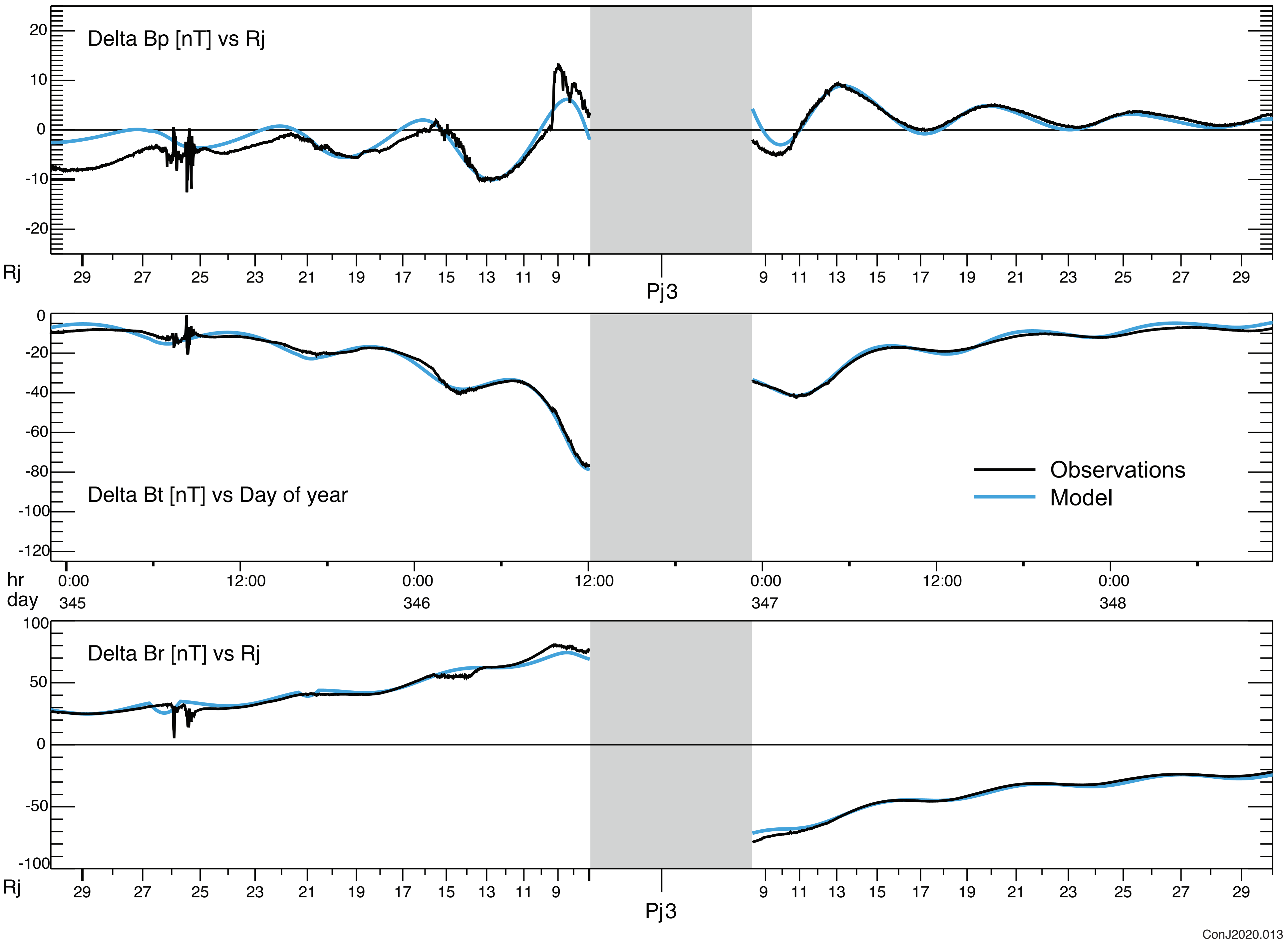
Delta Bp [nT] vs Rj
Delta Bt [nT] vs Day of year
Delta Br [nT] vs Rj
Observations
Model
Rj
Pj3
hr
day
0:00
12:00
345
346
347
348
ConJ2020.013

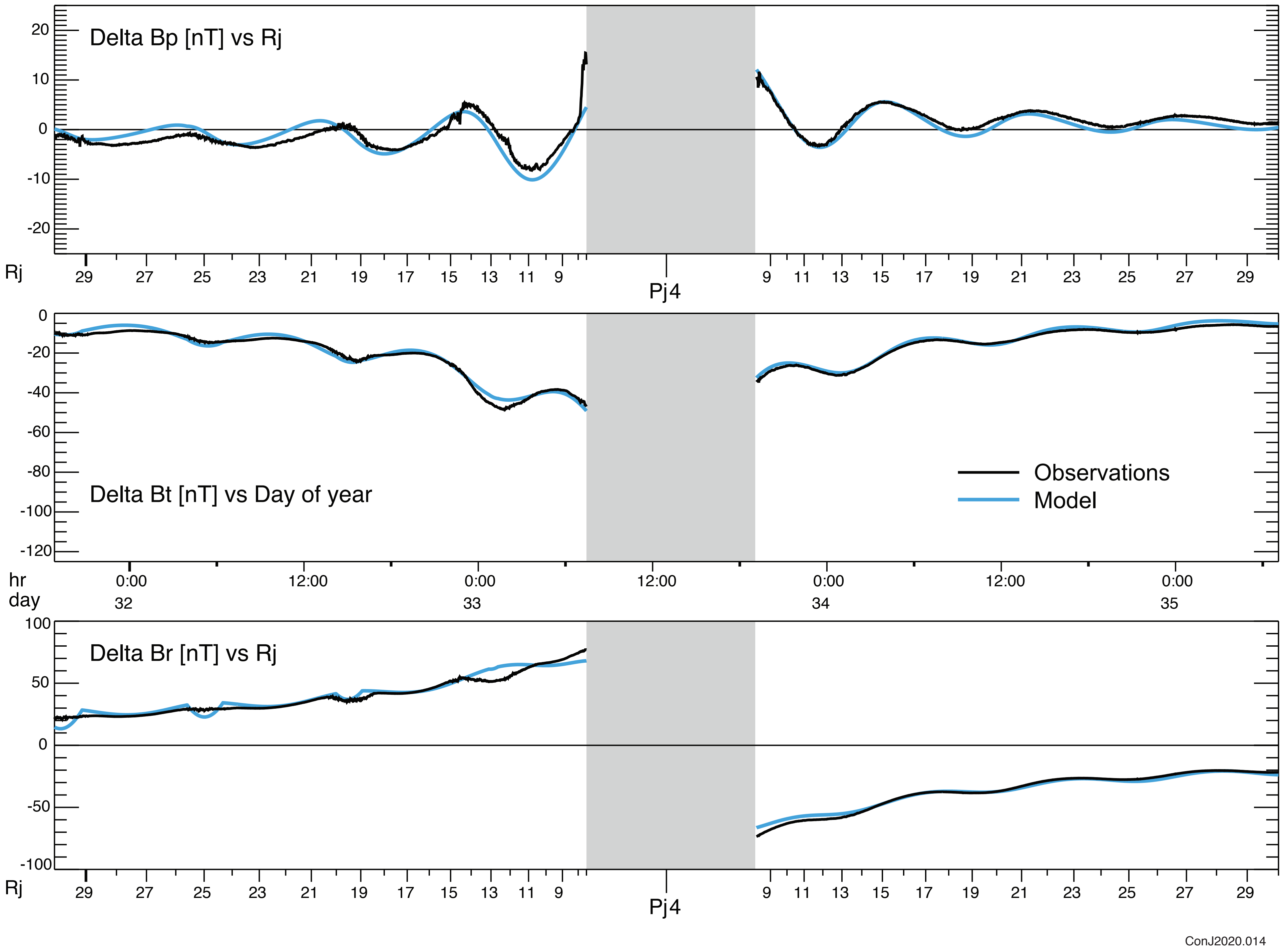

Delta Bp [nT] vs Rj
20
10
0
-10
-20
Rj
29
27
25
23
21
19
17
15
13
11
9
Pj4
9
11
13
15
17
19
21
23
25
27
29
0
-20
-40
-60
-80
-100
-120
Delta Bt [nT] vs Day of year
Observations
Model
hr
day
0:00
12:00
0:00
12:00
0:00
12:00
0:00
32
33
34
35
100
50
0
-50
-100
Delta Br [nT] vs Rj
Rj
29
27
25
23
21
19
17
15
13
11
9
Pj4
9
11
13
15
17
19
21
23
25
27
29
ConJ2020.014

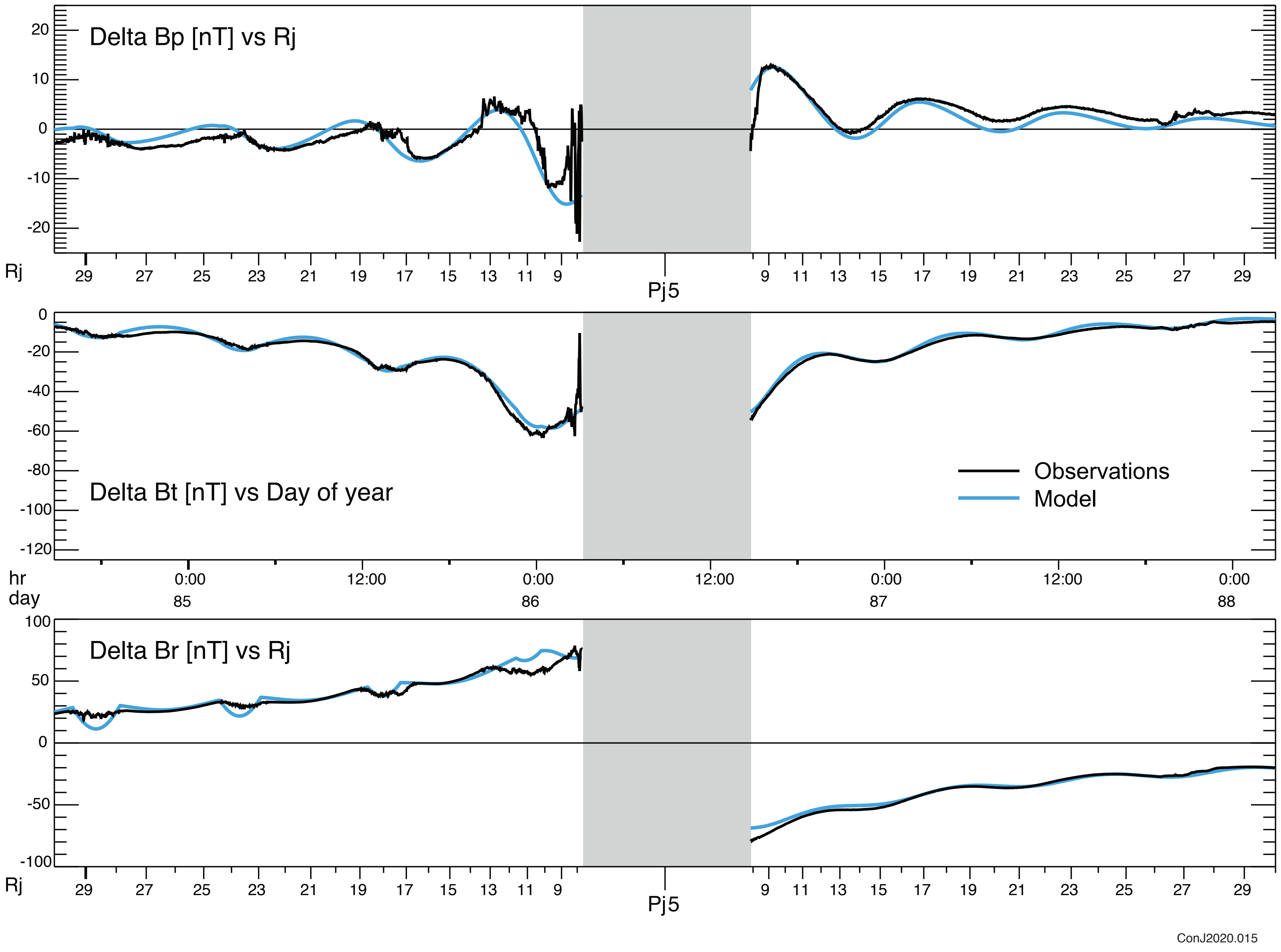

Delta Bp [nT] vs Rj
20
10
0
-10
-20
Rj
29 27 25 23 21 19 17 15 13 11 9
Pj5
9 11 13 15 17 19 21 23 25 27 29
Delta Bt [nT] vs Day of year
0
-20
-40
-60
-80
-100
-120
Observations
Model
hr
day
0:00 12:00 0:00 12:00 0:00 12:00 0:00
85 86 87 88
Delta Br [nT] vs Rj
100
50
0
-50
-100
ConJ2020.015

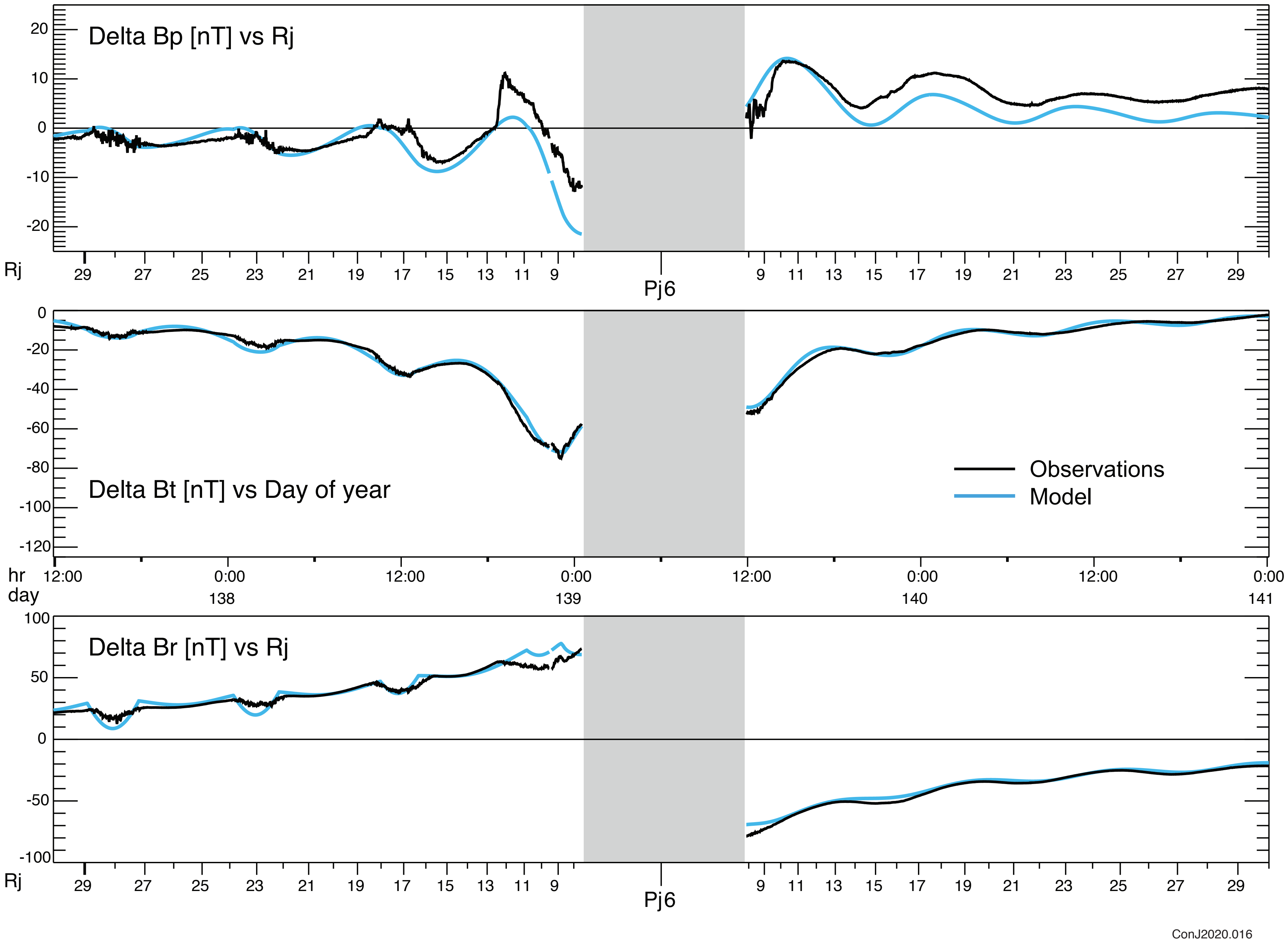

Delta Bp [nT] vs Rj
20
10
0
-10
-20
Rj
29 27 25 23 21 19 17 15 13 11 9
Pj6
9 11 13 15 17 19 21 23 25 27 29
Delta Bt [nT] vs Day of year
0
-20
-40
-60
-80
-100
-120
Observations
Model
hr
day
12:00 0:00 12:00 0:00 12:00 0:00 12:00 0:00
138 139 140 141
Delta Br [nT] vs Rj
100
50
0
-50
-100
Rj
29 27 25 23 21 19 17 15 13 11 9
Pj6
9 11 13 15 17 19 21 23 25 27 29
ConJ2020.016

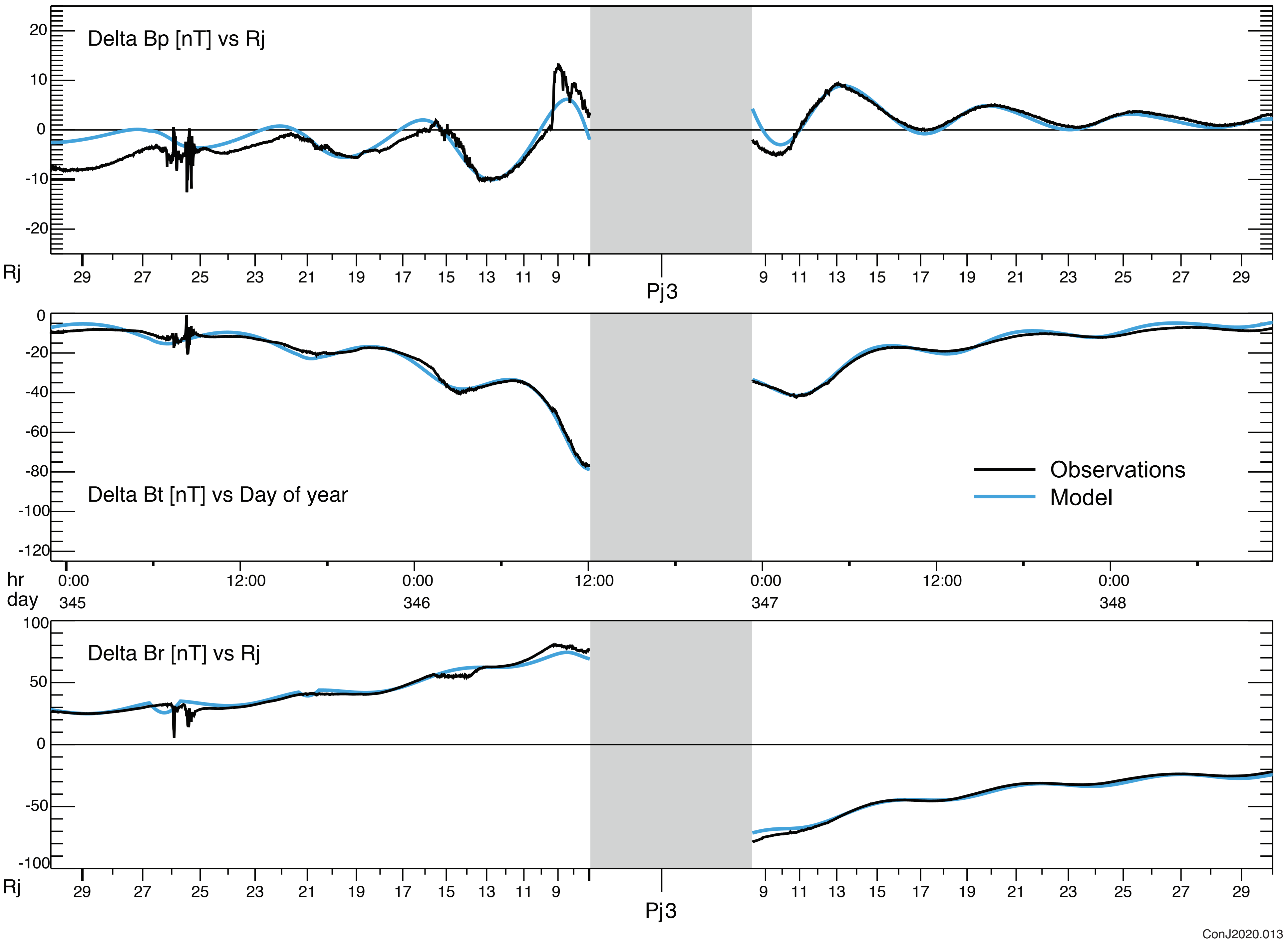
Delta Bp [nT] vs Rj
20
10
0
-10
-20
Rj
29 27 25 23 21 19 17 15 13 11 9
Pj3
9 11 13 15 17 19 21 23 25 27 29
Delta Bt [nT] vs Day of year
0
-20
-40
-60
-80
-100
-120
Observations
Model
hr
day
0:00 12:00 0:00 12:00 0:00 12:00 0:00
345 346 347 348
Delta Br [nT] vs Rj
100
50
0
-50
-100
Rj
29 27 25 23 21 19 17 15 13 11 9
Pj3
9 11 13 15 17 19 21 23 25 27 29
ConJ2020.013

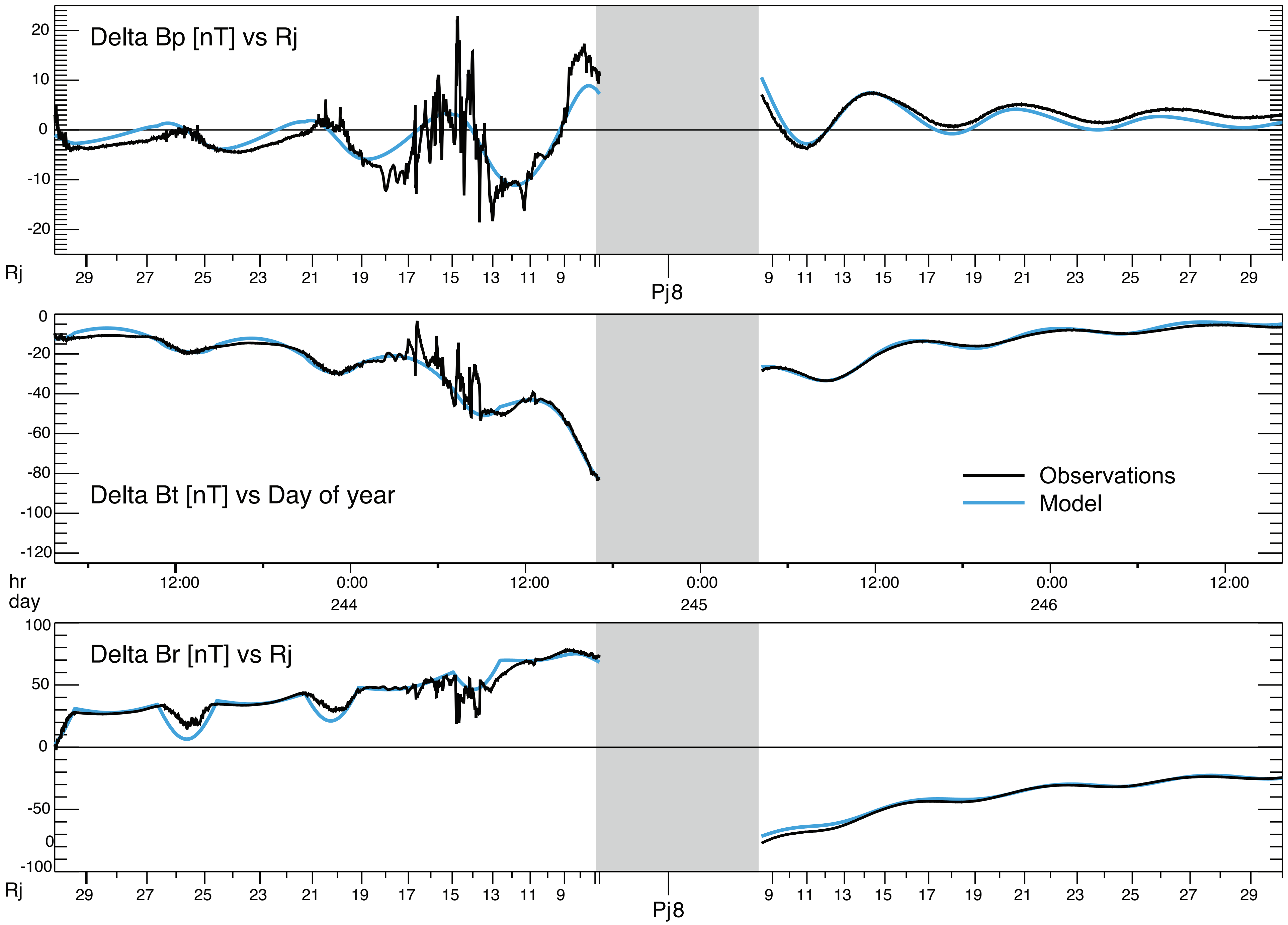

ConJ2020.018

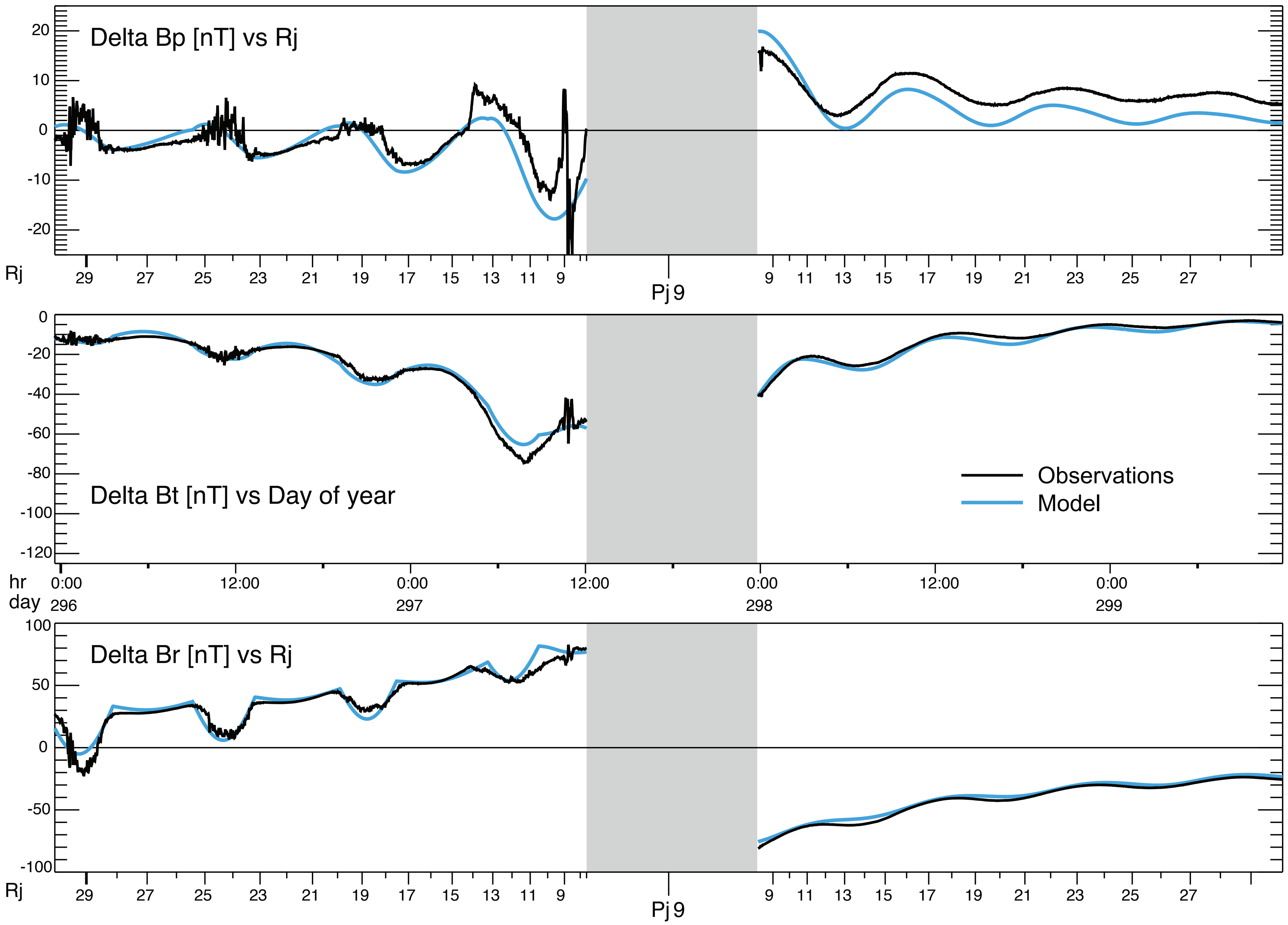

ConJ2020.019

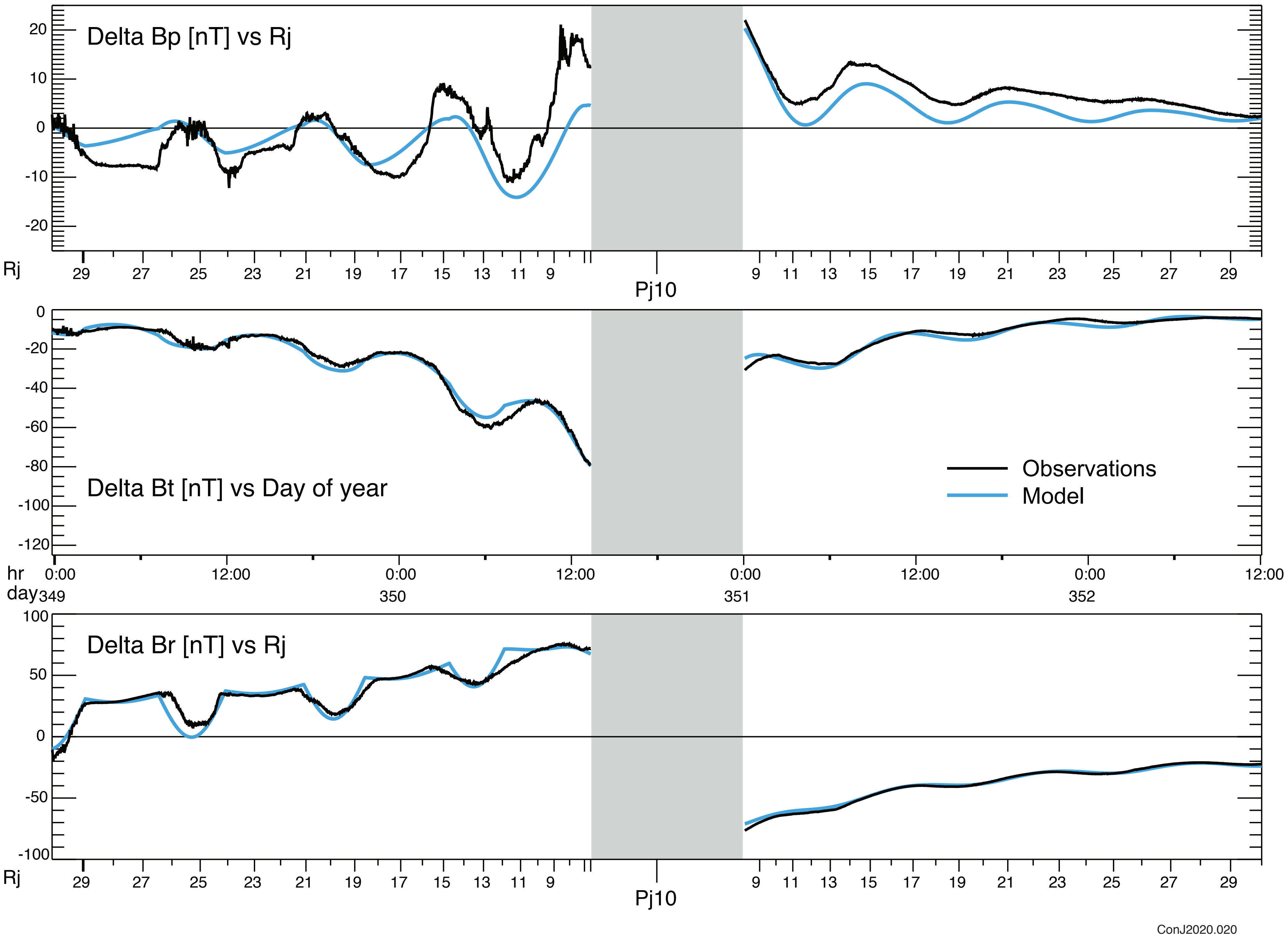
Delta Bp [nT] vs Rj
Delta Bt [nT] vs Day of year
Delta Br [nT] vs Rj
Observations
Model
Pj10
Rj
hr
day
0:00
12:00
349
350
351
352
ConJ2020.020

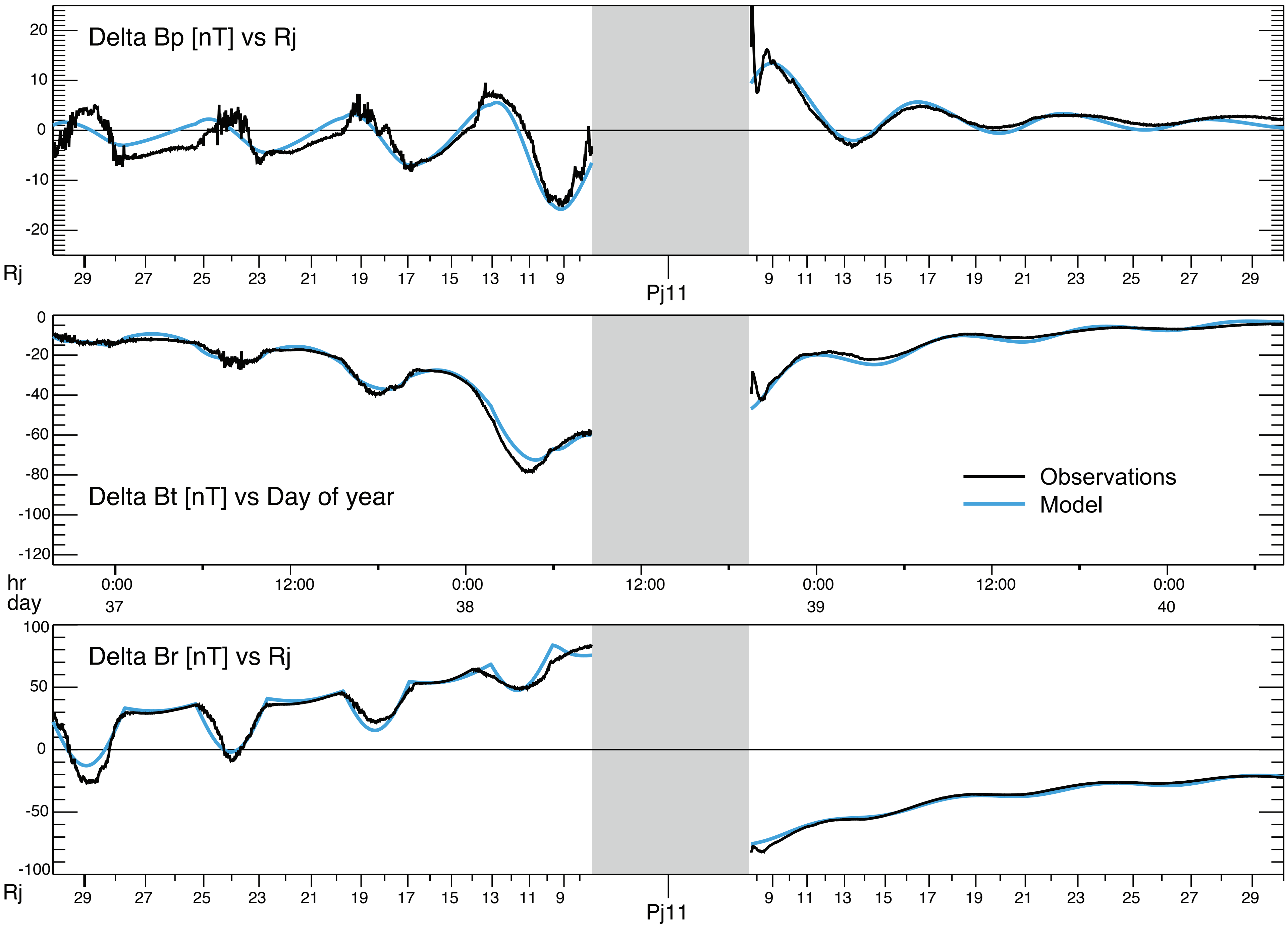

ConJ2020.021

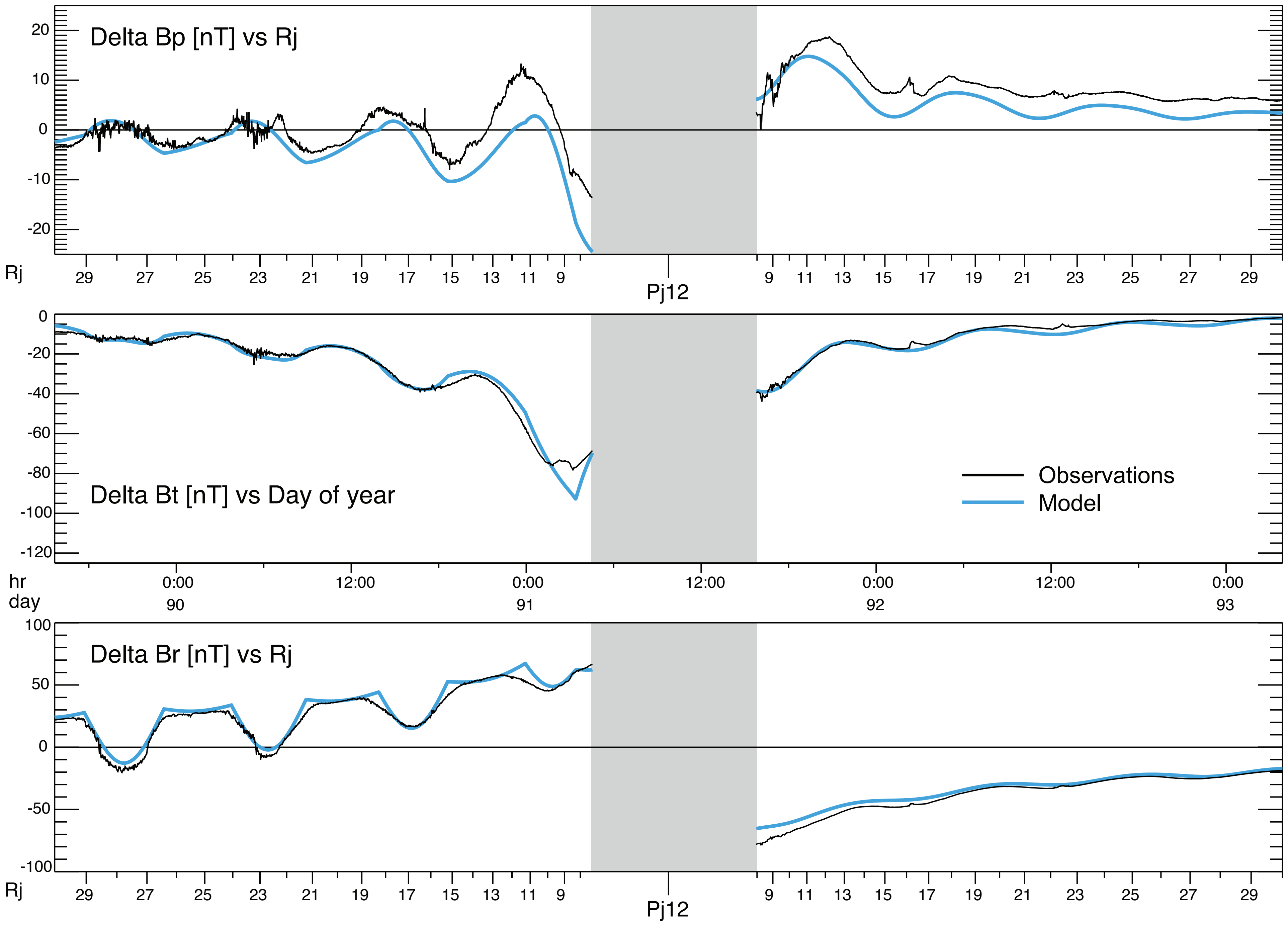

ConJ2020.022

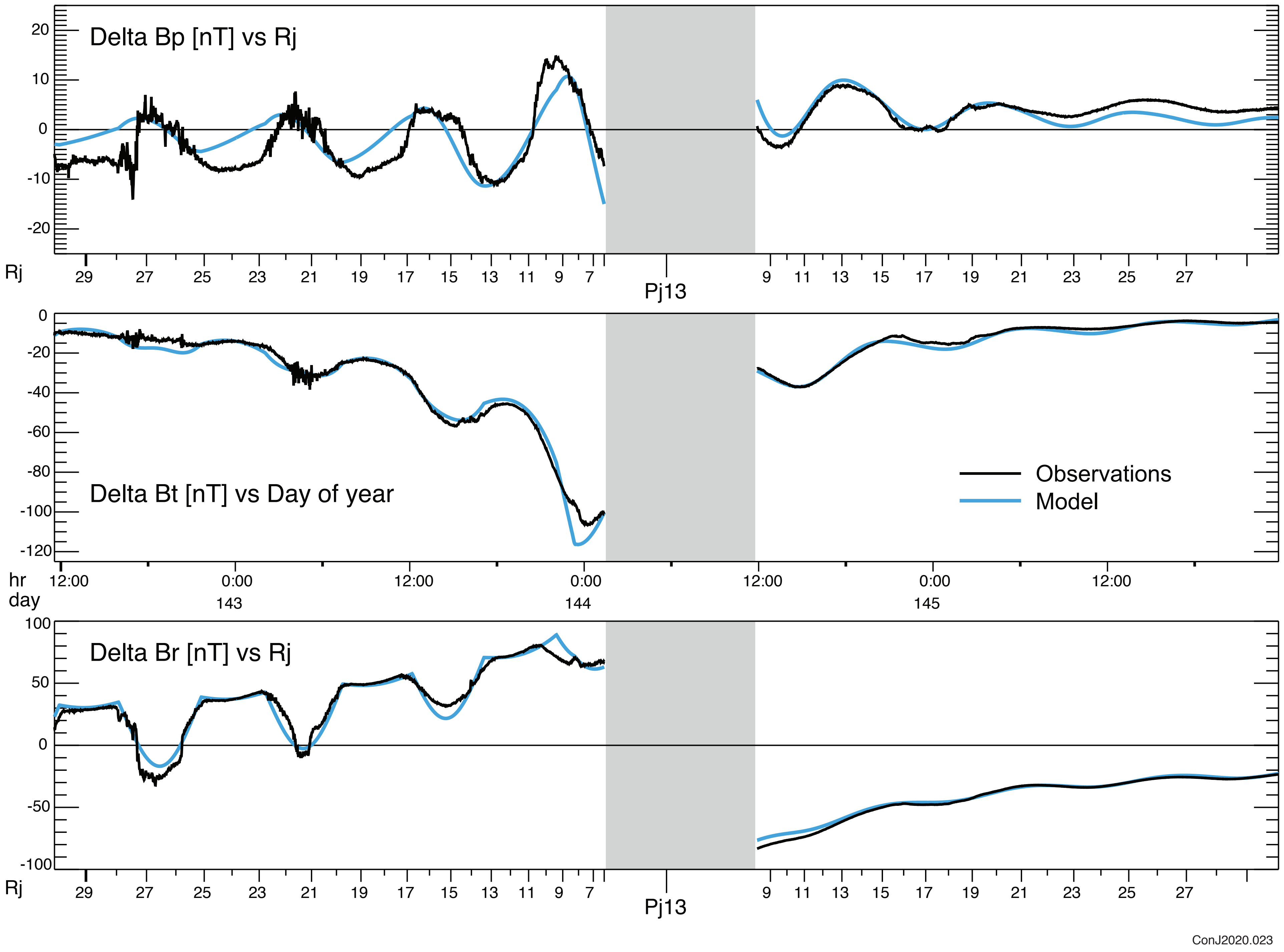

Delta Bp [nT] vs Rj
20
10
0
-10
-20
Rj
29
27
25
23
21
19
17
15
13
11
9
7
Pj13
9
11
13
15
17
19
21
23
25
27
Delta Bt [nT] vs Day of year
0
-20
-40
-60
-80
-100
-120
hr
day
12:00
0:00
143
12:00
0:00
144
12:00
0:00
145
12:00
Observations
Model
Delta Br [nT] vs Rj
100
50
0
-50
-100
Rj
29
27
25
23
21
19
17
15
13
11
9
7
Pj13
9
11
13
15
17
19
21
23
25
27
ConJ2020.023

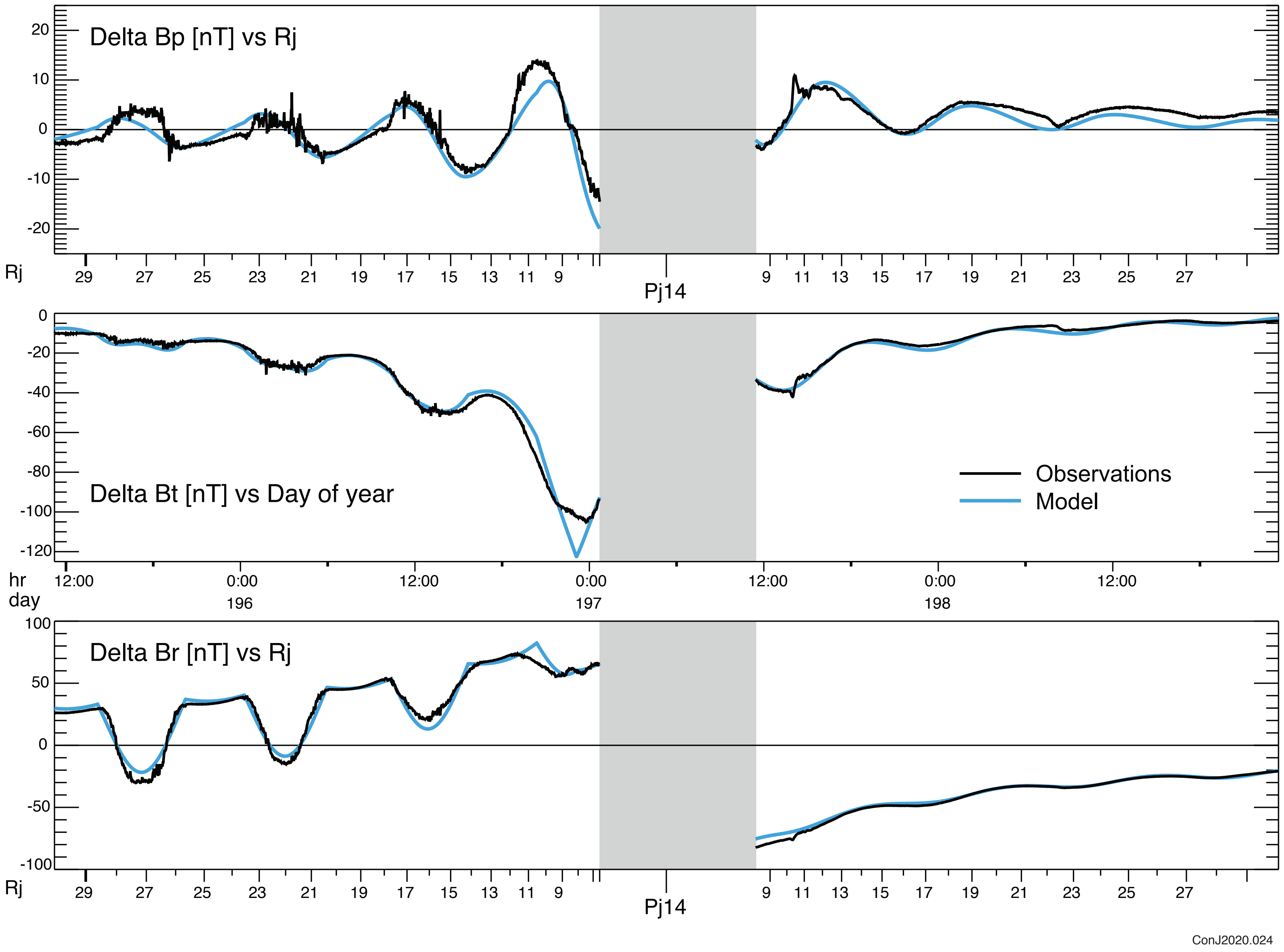

Delta Bp [nT] vs Rj
20
10
0
-10
-20
Rj
29
27
25
23
21
19
17
15
13
11
9
Pj14
9
11
13
15
17
19
21
23
25
27
Delta Bt [nT] vs Day of year
0
-20
-40
-60
-80
-100
-120
Observations
Model
hr
day
12:00
0:00
196
12:00
0:00
197
12:00
0:00
198
12:00
Delta Br [nT] vs Rj
100
50
0
-50
-100
Rj
29
27
25
23
21
19
17
15
13
11
9
Pj14
9
11
13
15
17
19
21
23
25
27
ConJ2020.024

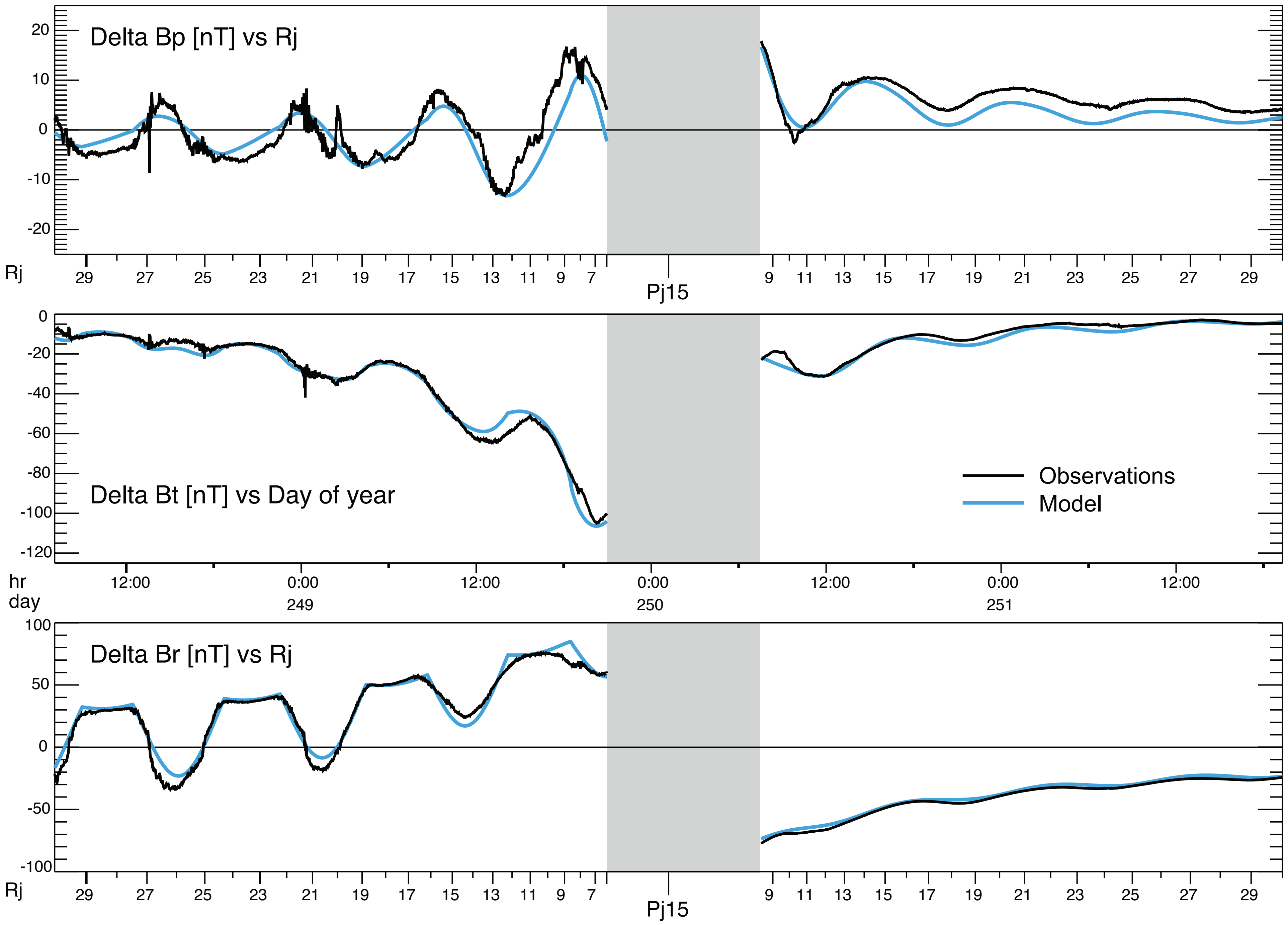

ConJ2020.025

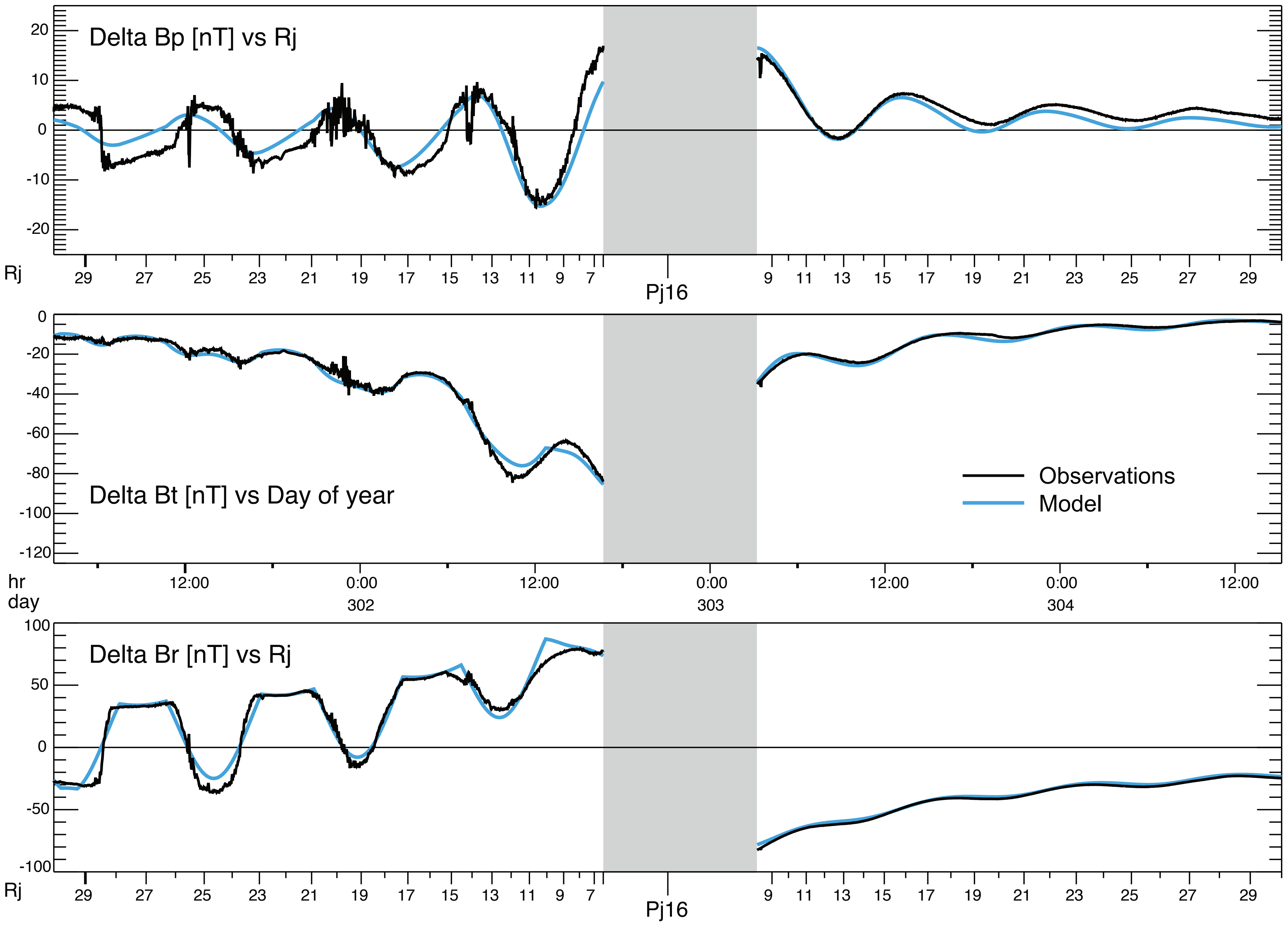

ConJ2020.026

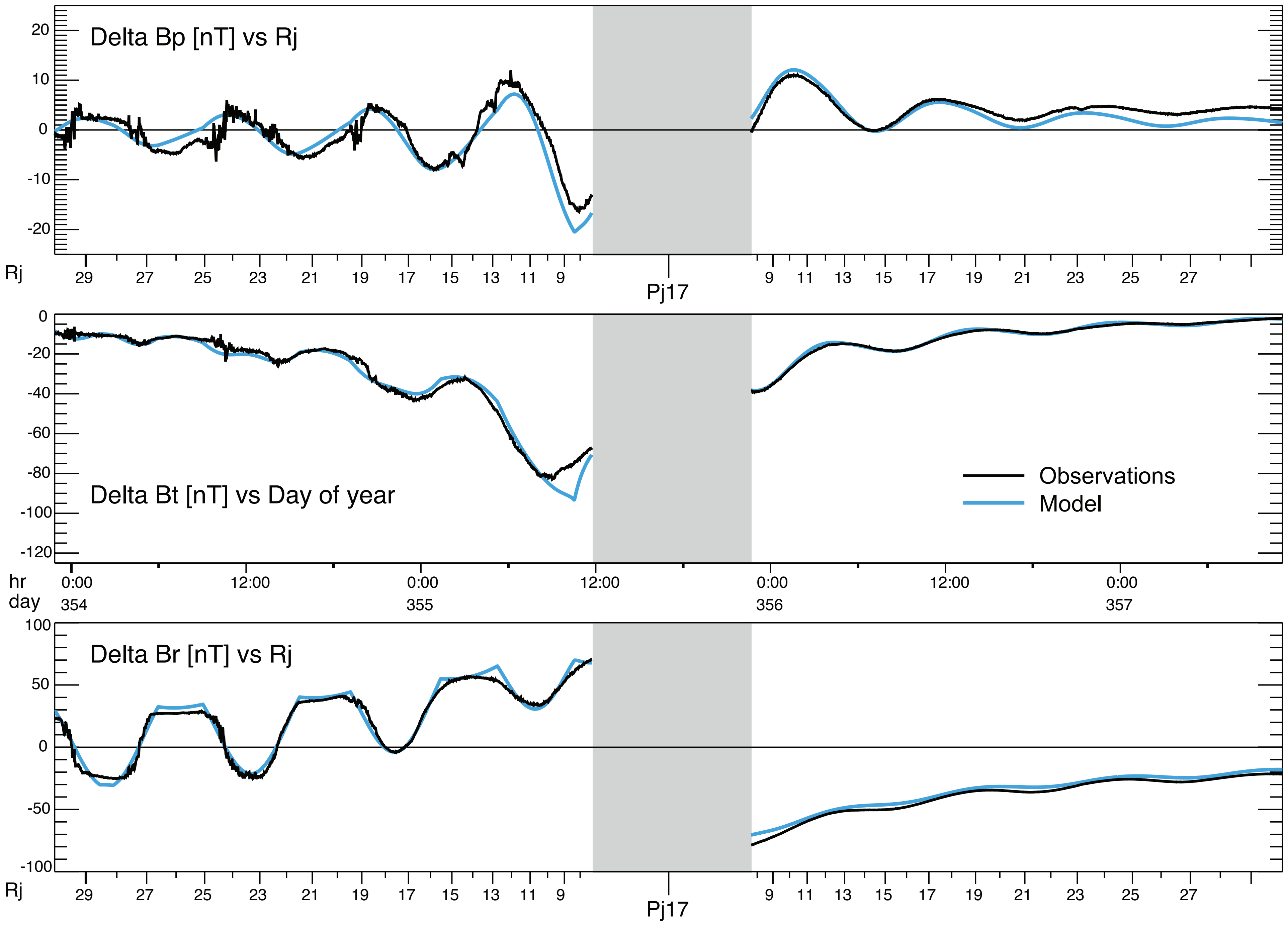

ConJ2020.027

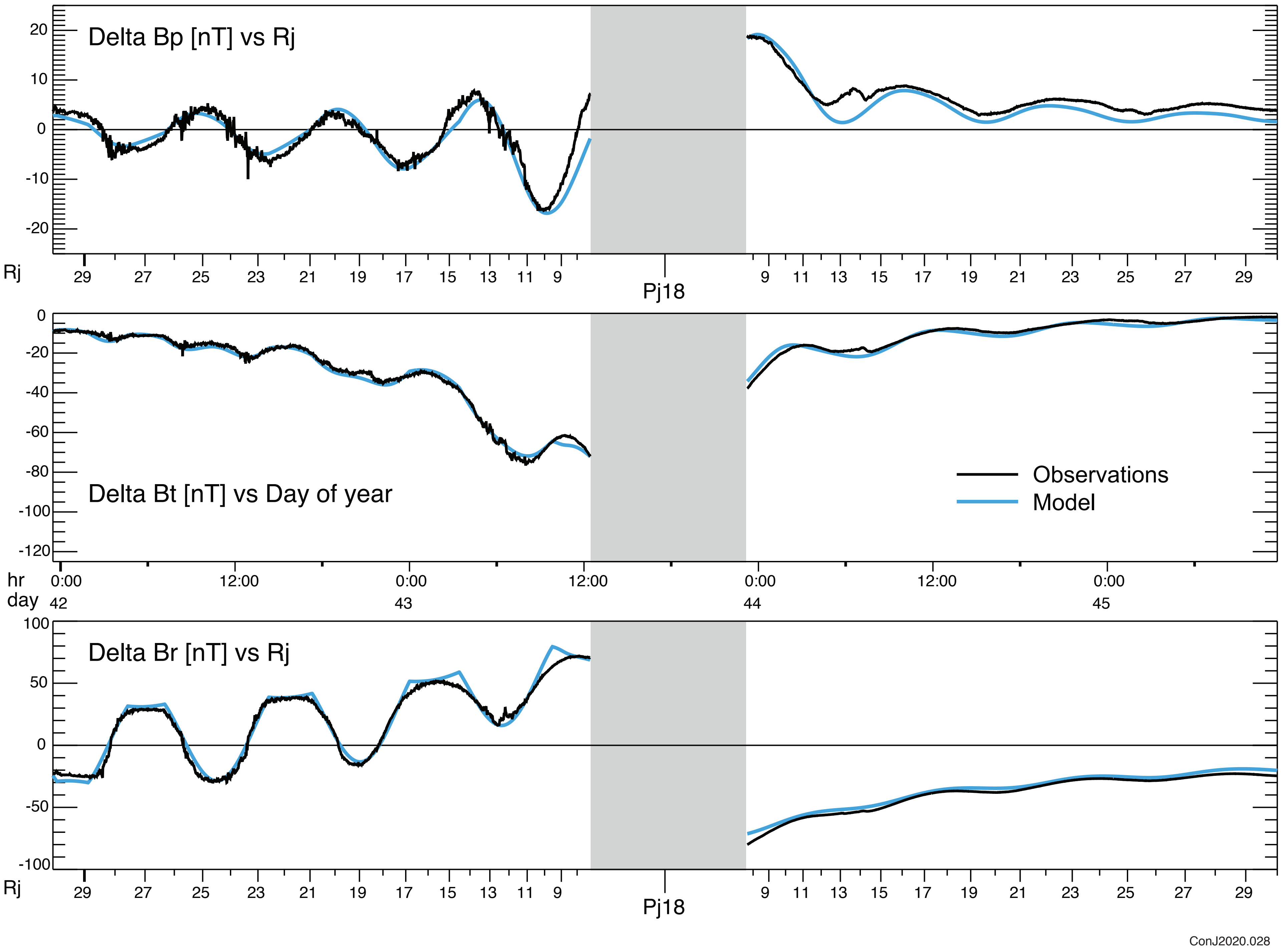

Delta Bp [nT] vs Rj
20
10
0
-10
-20
Rj
29 27 25 23 21 19 17 15 13 11 9
Pj18
9 11 13 15 17 19 21 23 25 27 29
0
-20
-40
-60
-80
-100
-120
Delta Bt [nT] vs Day of year
Observations
Model
hr 0:00 12:00 0:00 12:00 0:00 12:00 0:00
day 42 43 44 45
Delta Br [nT] vs Rj
100
50
0
-50
-100
Rj
29 27 25 23 21 19 17 15 13 11 9
Pj18
9 11 13 15 17 19 21 23 25 27 29
ConJ2020.028

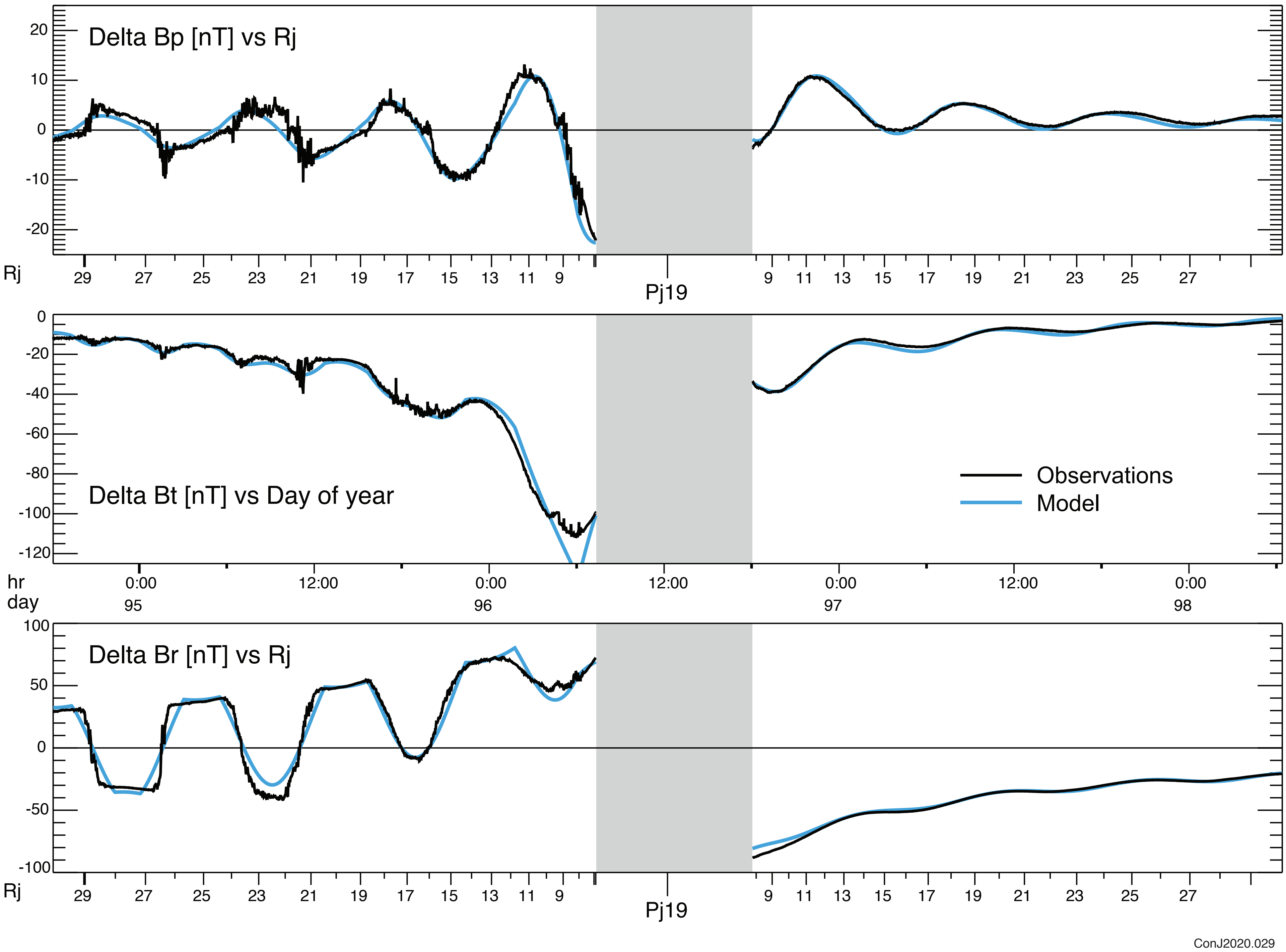

Delta Bp [nT] vs Rj
Rj
Pj19
Delta Bt [nT] vs Day of year
Observations
Model
hr
day
Delta Br [nT] vs Rj
ConJ2020.029

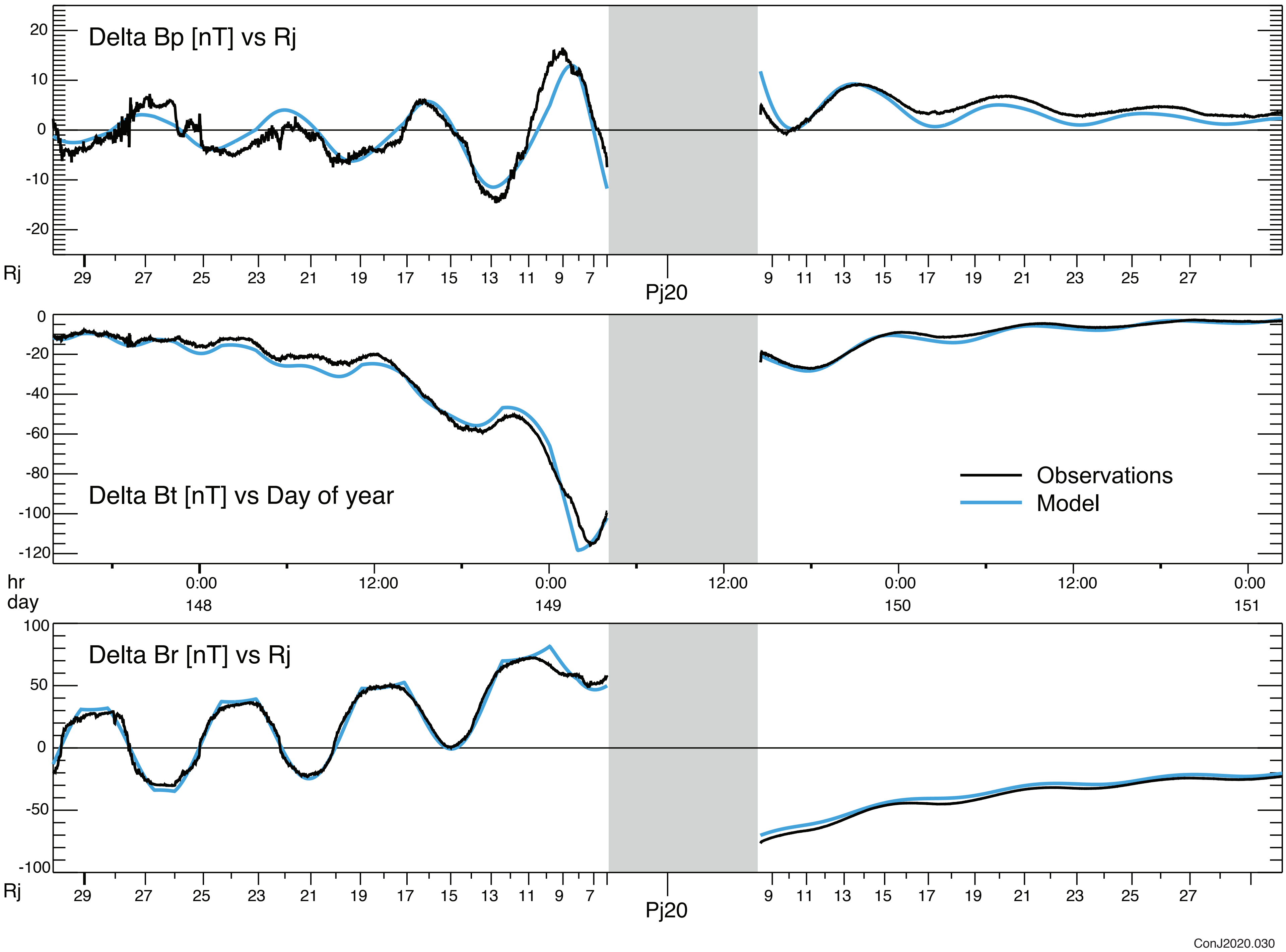
Delta Bp [nT] vs Rj
20
10
0
-10
-20
Rj
29 27 25 23 21 19 17 15 13 11 9 7
Pj20
9 11 13 15 17 19 21 23 25 27
0
-20
-40
-60
-80
-100
-120
Delta Bt [nT] vs Day of year
Observations
Model
hr
day
0:00 12:00 0:00 12:00 0:00 12:00 0:00
148 149 150 151
100
50
0
-50
-100
Delta Br [nT] vs Rj
Rj
29 27 25 23 21 19 17 15 13 11 9 7
Pj20
9 11 13 15 17 19 21 23 25 27
ConJ2020.030

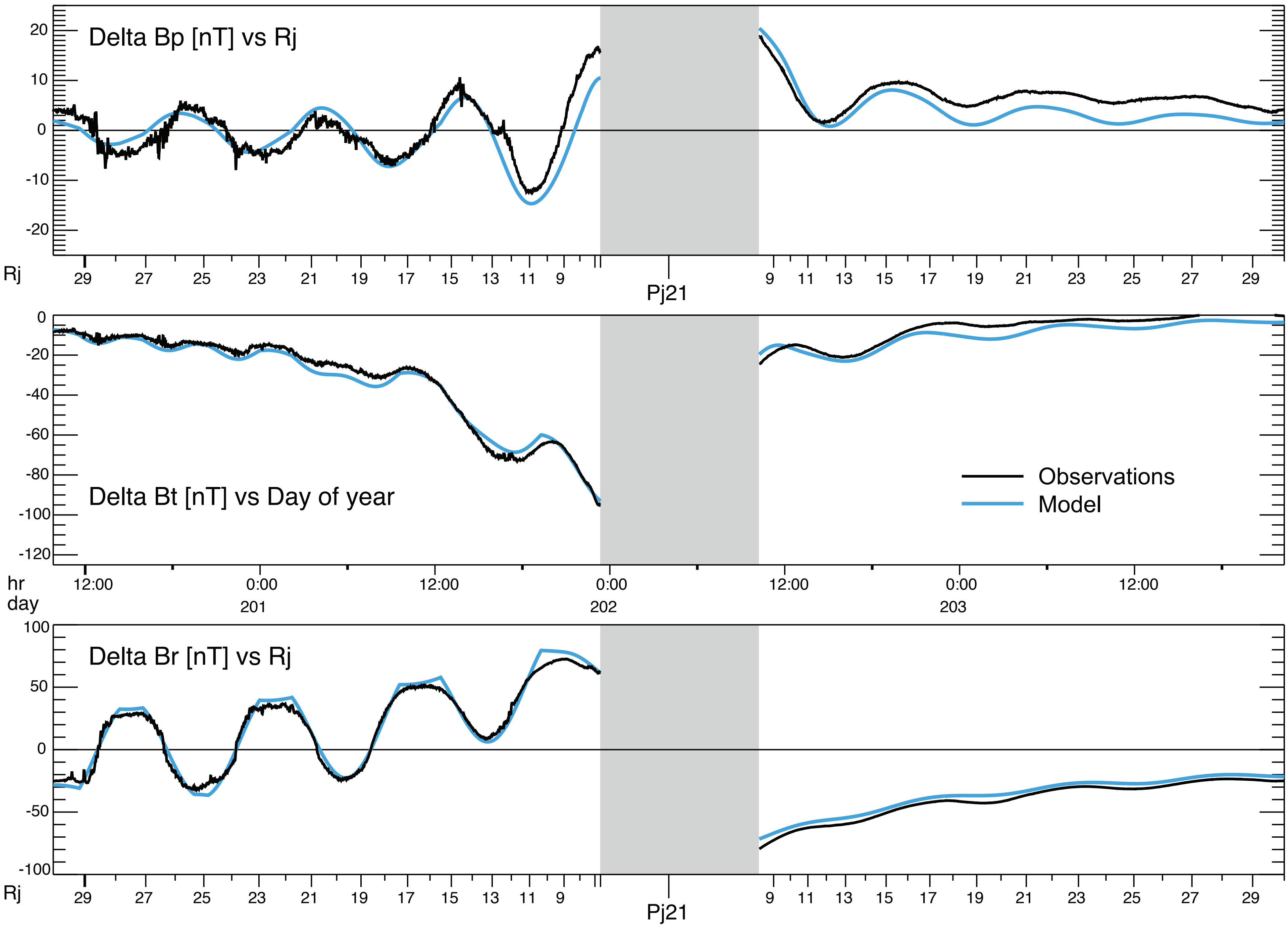

ConJ2020.031

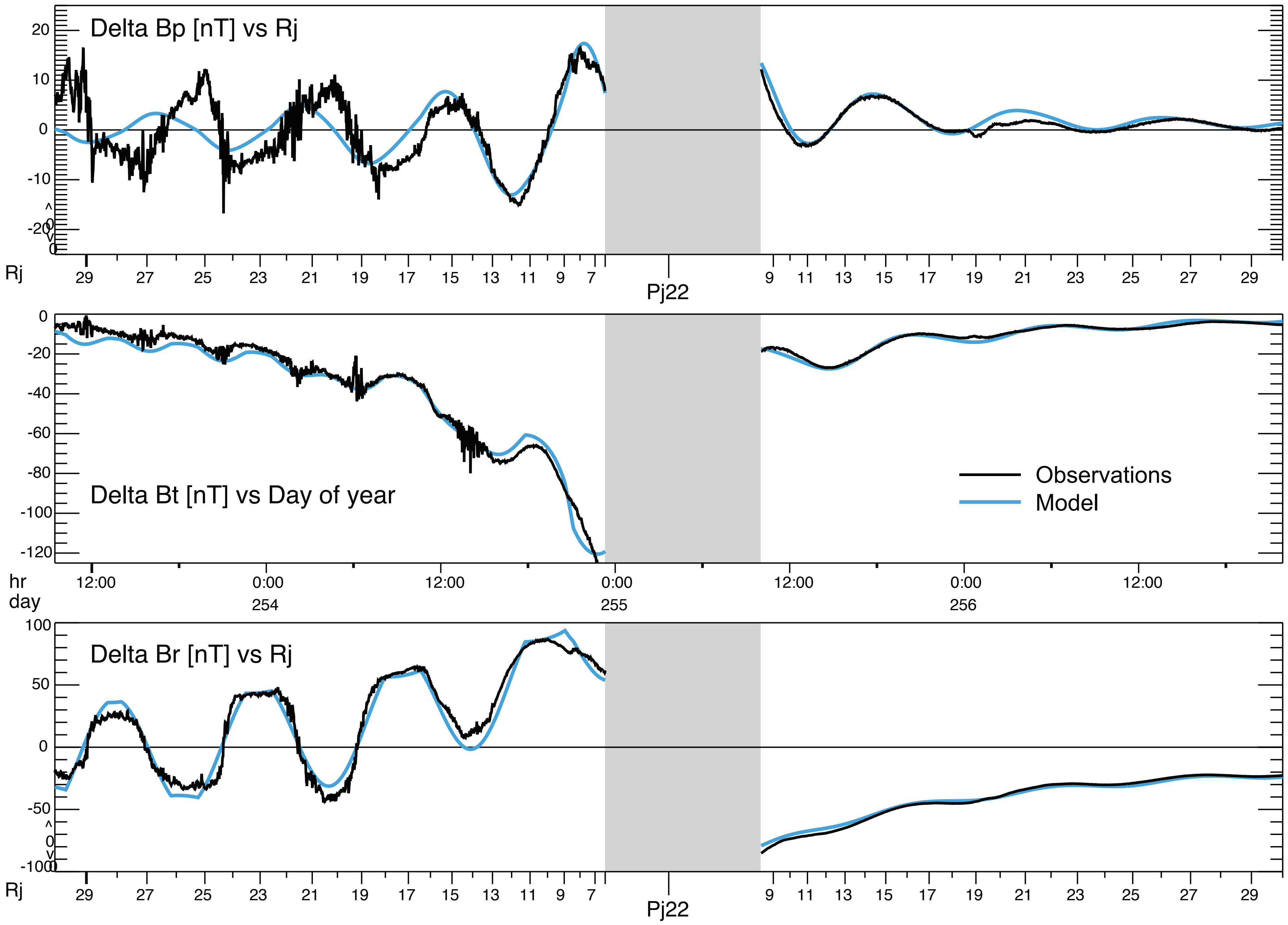

ConJ2020.032

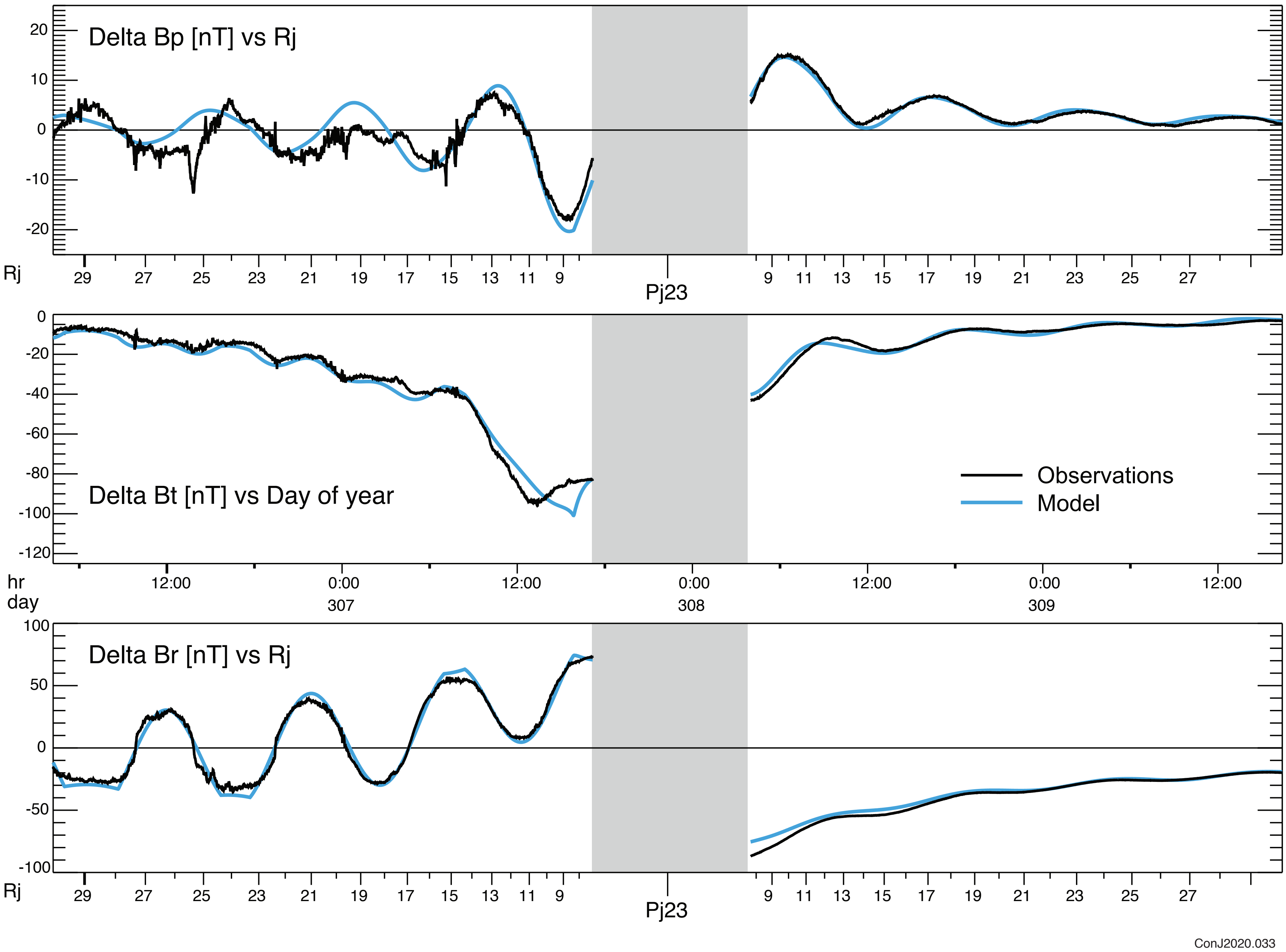
Delta Bp [nT] vs Rj
Delta Bt [nT] vs Day of year
Delta Br [nT] vs Rj
Observations
Model
Pj23
Rj
hr
day
12:00
0:00
307
308
309
ConJ2020.033

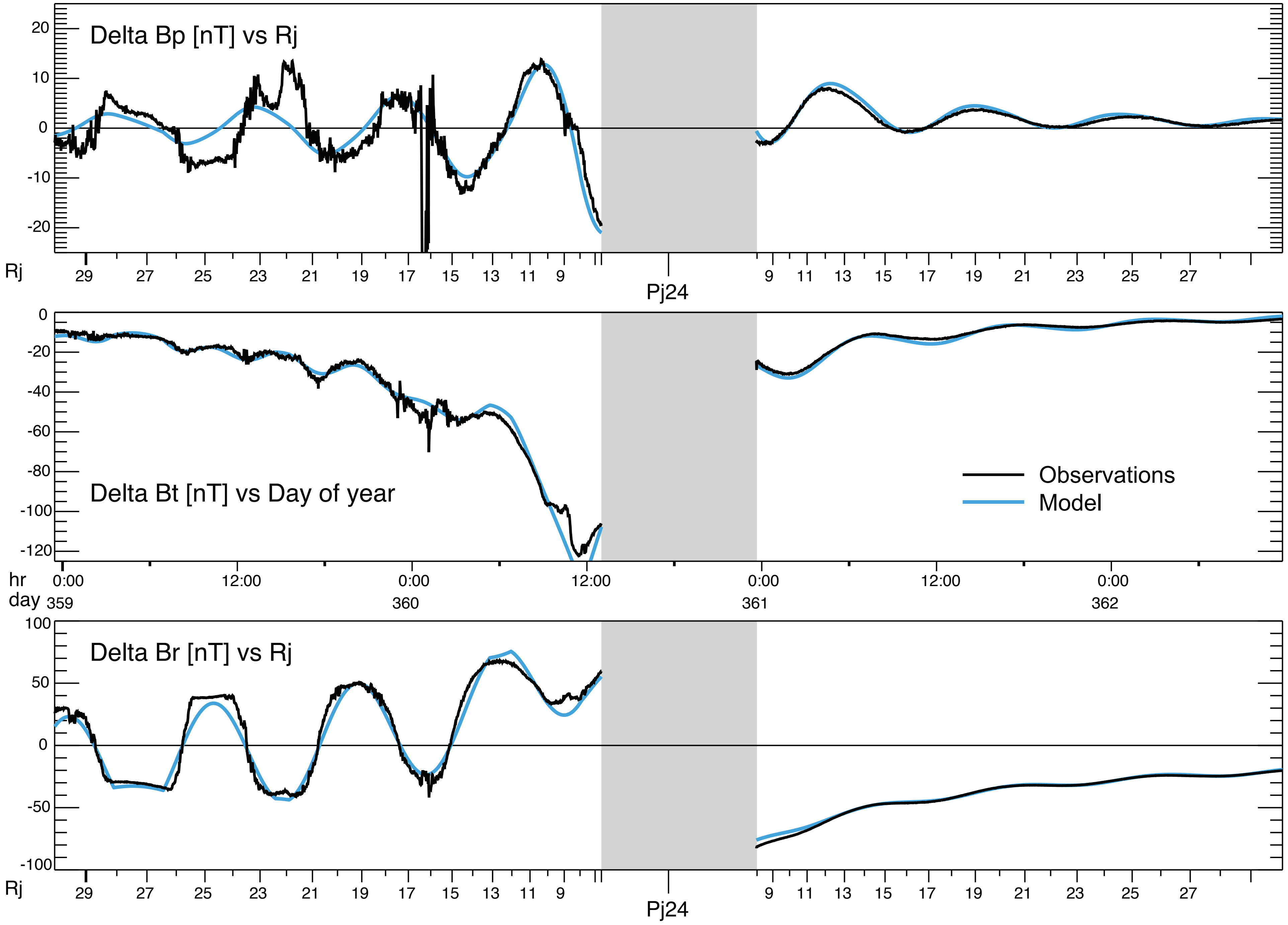

ConJ2020.034
